# Genomic revelations: investigating rotavirus a presence in wild ruminants and its zoonotic potential

**DOI:** 10.3389/fvets.2024.1429654

**Published:** 2024-08-15

**Authors:** Petra Šenica, Diana Žele Vengušt, Gorazd Vengušt, Urška Kuhar

**Affiliations:** ^1^Veterinary Faculty, Institute of Microbiology and Parasitology, University of Ljubljana, Ljubljana, Slovenia; ^2^Veterinary Faculty, Institute of Pathology, Wild Animals, Fish and Bees, University of Ljubljana, Ljubljana, Slovenia

**Keywords:** rotaviruses, wild ruminants, zoonotic potential, phylogenetic analysis, genomic diversity, cross-species infection

## Abstract

**Introduction:**

Rotaviruses A (RVA) are a major cause of acute viral gastroenteritis in humans worldwide and are responsible for about two million hospitalizations per year. They can also infect other mammals such as pigs, calves, goats, lambs, and horses, in which they are also considered a major cause of viral diarrhea. While RVA is well studied in humans and domestic animals, its occurrence in wild ruminants is not well known. The RVA genome is a double-stranded RNA consisting of 11 segments, and genotyping is based on the VP7 (G) and VP4 (P) segments. Currently, there are 42G genotypes and 58P genotypes. RVA has a high mutation rate, and some combinations of G and P genotypes can infect different animal species, leading to speculation about the potential for zoonotic transmission.

**Materials and methods:**

A total of 432 fecal samples were collected from roe deer, red deer, chamois, mouflon and Alpine ibex in Slovenia between 2017 and 2021. To investigate the presence of RVA in wild ruminants, real-time RT-PCR was used. Positive samples were subjected to next generation sequencing (NGS) using RIP-seq method.

**Results and discussion:**

In total, 7 samples were RVA positive. Complete genomes were determined and phylogenetically analyzed for all 7 RVAs. Four different genotype constellations were present in 7 positive RVA animals: G8-P[14]-I2- R2-C2-M2-A3-N2-T6-E2-H3, G6-P [14]-I2-R2-C2-M2-A11-N2-T6-E2-H3, G10-P [15]-I2-R2-C2-M2-A3-N2-T6-E2-H3 and G10-P [15]-I2-R2-C2-M2-A11- N2-T6-E2-H3. Genotypes G6P[14] and G10P[15] were found in both roe deer and red deer, representing the first confirmed occurrence of RVA in red deer. In addition, genotype G8P[14] was found in chamois, representing the first known case of positive RVA in this species. Some of these genotypes have also been found in humans, indicating the potential for zoonotic transmission.

## 1 Introduction

Rotaviruses (RVs) are the most common cause of acute viral gastroenteritis in humans worldwide and are responsible for 2 million hospitalizations per year. Symptoms are most common in young children and immunocompromised people. In animals, such as pigs (1, 2), cattle (2), goats (3), sheep (3), and horses (4), rotaviruses are commonly diagnosed in animals with viral diarrhea. Rotaviruses can also infect other mammals and birds (5). The infection is via the fecal-oral route or through direct contact. The zoonotic potential of some RVs is suggested by the identification of unusual human RV strains with characteristics more commonly found in animals (6). There are several types of RVs: Rotavirus A (RVA), Rotavirus B (RVB), Rotavirus C (RVC) and Rotavirus H (RVH), which infect humans and various animals, while Rotavirus D (RVD), Rotavirus E (RVE), Rotavirus F (RVF) and Rotavirus G (RVG) have only been found in animals, mainly birds (7). The most important species from an epidemiological point of view for infections in humans and animals is RVA (8). RV belong to the Reoviridae family with genome that consists of 11 dsRNA molecules, with a total length of approximately 18,500 base pairs. These RNA segments encode six structural proteins (VP1, VP2, VP3, VP4, VP6, and VP7) and six non-structural proteins (NSP1 to NSP6). Each RNA segment is monocistronic, except for segment 11, which, in certain strains, contains two overlapping open reading frames (ORFs) encoding NSP5 and NSP6 (9, 10). Two outer layer capsid proteins (VP7 and VP4) are used for RVA classification into G and P genotypes. Currently, 42 G- genotypes and 58P- genotypes are known (11). Some combinations of G and P genotypes are typical for certain animals; for example, the G8 P[14] genotype is responsible for rotaviral infections in cattle, but this genotype has also been found in humans. This is one of the reasons why it is suspected that rotaviruses are transmitted zoonotically (12). The segmented genome of RV facilitates reassortment, i.e., the exchange of gene segments, leading to the emergence of unique strains with novel genomic constellations derived from two-parent RV strains, as frequently observed in human- animal strains. Although RVs generally favor selective hosts, interspecies transmission has been observed several times. Recently, new genotypes have been identified in different animal species, suggesting an origin in multiple hosts (13, 14). The complete genome analysis allows the determination of genotypes for all 11 segments using the abbreviations Gx-P[x]-Ix- Rx-Cx-Mx-Ax-Nx-Tx-Ex-Hx developed by the Rotavirus Classification Working Group (RCWG) (15). Based on the constellation of all 11 segments, there are 3 main genogroups of RVA found in humans and animals, namely Wa-like (Gx-P[x]-I1-R1-C1-M1-A1-N1-T1-E1-H1) DS-1-like (Gx-P[x]-I2-R2-C2-M2-A2-N2-T2-E2-H2) and AU-1-like (Gx-P[x]-I3–R3–C3–M3–A3–N3–T3–E3–H3). Wa-like and DS-1-like are the most common genogroups found in humans. However, the differences lie not only in the genetic constellation of the 11 segments, but also which animals can be infected. Wa-like human RVAs are very similar to porcine RVAs, DS -1-like to bovine RVAs and AU-1-like to canine and feline RVAs. The question therefore arises as to whether these RVAs are of animal origin and become zoonotic viruses (15–17).

The aim of our study was to detect RVAs in the intestinal tract of wild ruminants and to investigate the potential for interspecies transmission of RVAs between wild ruminants and other animals and humans and whether potentially found RVAs could be of zoonotic origin.

## 2 Materials and methods

### 2.1 Sample collection and preparation

Fecal samples from 432 wild ruminants (249 roe deer, 93 red deer, 78 chamois, 10 mouflon, and 2 ibex) were collected during 2017 and 2021 in the territory of Slovenia, Europe. Samples were obtained as a part of national wildlife passive health surveillance program. Hunters and professional game wardens from all over the country were encouraged to provide carcasses of wild ruminants through various information channels (hunter magazine, administrative services of hunter organizations). Wild ruminants were either found dead in the wild or legally harvested because animals exhibited clinical signs or the animals were harvested during the regular annual culling. Carcasses were submitted to the Veterinary Faculty, University of Ljubljana. Fecal samples were prepared in a 1:4 ratio (1 part fecal sample and 4 parts RPMI medium) followed by centrifugation at 2,500 rpm for 15 min. The supernatant was collected and centrifuged at 14,000 rpm for 10 min. Subsequently, the supernatant was collected and 5 prepared samples were pooled proportionally for further analysis.

### 2.2 RNA extraction and molecular detection

Total RNA was extracted with the MagMAX CORE Nucleic Acid Purification Kit on the KingFisher Flex System (Thermo Fisher Scientific, Carlsbad, United States). The extracted RNA was first denatured at 95°C for 5 min, and then real-time RT-PCR analysis was performed on isolated pooled RNA samples using the commercial kit qScript XLT one step RT -gPCR ToughMix, ROX (Quantabio, United States) with previously used 5 forward, 2 reverse primers and MGB probe (18). Individual samples from positive pools were analyzed with the real-time RT-PCR assay as described above.

### 2.3 NGS, bioinformatic analysis and complete genome construction

RNA from 7 rotavirus-positive samples (4 roe deer, 2 red deer and 1 chamois) was isolated using Trizol reagent (Invitrogen, United States) in combination with MaXtract High Density Tubes 2 mL (Qiagen, Germany) according to the manufacturer’s instructions. The isolated RNA was sent to Novogene Ltd. (United Kingdom) for RNA immunoprecipitation sequencing (RIP-seq) (19) using Illumina technology. The sequenced reads were trimmed using BBduk v.38.84 as a plugin in Geneious Prime software suite v. 2022.1.1 (Biomatters Ltd., Auckland, New Zealand) and then *de novo* assembled using metaSPAdes v.3.13.1 (20) to generate contigs. These contigs were taxonomically classified using Diamond v.0.9.24 (21) with integrated Blast database v.2.9.0 and visualized using MEGAN6 software v.6.17.0 (22). The fasta formats of the contigs belonging to the genus RVA were extracted from MEGAN software and imported into Geneious Prime software suite v. 2022.1.1 (Biomatters Ltd., Auckland, New Zealand), which was used for further downstream bioinformatic analyses. The contigs were mapped to the reference sequences from GenBank based on blastn search (Supplementary Tables S2–S8) and then whole genomes were created. To fill in the gaps in the genome sequences, primers were constructed for RT-PCR using primer design tool in Geneious Prime software and RT-PCR products were sent to Macrogen Europe for Sanger sequencing. To finalize the whole genomes and calculate genome coverage, trimmed reads and Sanger sequences from RT-PCR products were mapped onto the assembled genomes. The genome constellation was determined using the Subspecies Classification for Rotavirus A tool from the website BV-BRC (23).

### 2.4 Phylogenetic analysis and tree construction

For phylogenetic analysis, we selected 35 RVA genomes from GenBank relevant to the 7 RVA strains identified in our study, according to genotype constellations and including the closest relatives based on Blastn search of each segment (Table 1). The alignment of all 11 segments was performed using the MAFFT algorithm v.7.490 (24). The nucleotide identities were calculated based on the alignment. After alignment, phylogenetic trees for all 11 segments were constructed in MEGA11 software v.11.0.13 (25) using the maximum likelihood method based on Kimura’s 2-parameter model. One thousand replicates were used in the bootstrap analysis to calculate the branch statistics.

**Table 1:** Comparison of the genotype constellation of seven complete RVA genome sequences of Slovenian wild ruminants (bolded in text) with 35 relevant RVA genome sequences from GenBank.

Description	Origin	VP7 G	VP4 [P]	VP6 I	VP1 R	VP2 C	VP3 M	NSP1 A	NSP2 N	NSP3 T	NSP4 E	NSP5 H
RVA/Cow/−wt/JPN/Tottori-SG/2013/G15P[14]	Bo	15	14	2	2	2	2	3	2	6	2	3
RVA/Human-wt/BEL/B4106/2000/G3P[14]	Hu	3	14	2	2	2	3	9	2	6	5	3
RVA/Rabbit-tc/ITA/30–96/1996/G3P[14]	Rab	3	14	2	2	2	3	9	2	6	5	3
RVA/Human-wt/BEL/B1711/2002/G6P[6]	Hu	6	6	2	2	2	2	2	2	2	2	2
RVA/Human-tc/AUS/MG6/1993/G6P[14]	Hu	6	14	2	2	2	2	11	2	6	2	3
RVA/Human-wt/HUN/Hun5/1997/G6P[14]	Hu	6	14	2	2	2	2	11	2	6	2	3
RVA/Human-wt/BEL/B10925/1997/G6P[14]	Hu	6	14	2	2	2	2	3	2	6	2	3
RVA/Human-tc/ITA/PA169/1988/G6P[14]	Hu	6	14	2	2	2	2	3	2	6	2	3
RVA/Human-wt/ITA/111–05-27/2005/G6P[14]	Hu	6	14	2	2	2	2	3	2	6	2	3
RVA/Sheep-tc/ESP/OVR762/2002/G8P[14]	Ov	8	14	2	2	2	2	11	2	6	2	3
RVA/Human-tc/USA/Se584/1998/G6P[9]	Hu	6	9	2	2	2	2	3	2	1	2	3
RVA/Guanaco-wt/ARG/Chubut/1999/G8P[14]	Gua	8	14	2	5	2	2	3	2	6	12	3
RVA/Cow-wt/ARG/B383/1998/G15P[11]	Bo	15	11	2	5	2	2	13	2	6	12	3
RVA/Human-wt/HUN/BP1879/2003/G6P[14]	Hu	6	14	2	2	2	2	11	2	6	2	3
RVA/Human-wt/HUN/BP1062/2004/G8P[14]	Hu	8	14	2	2	2	2	11	2	6	2	3
**RVA/Chamois-wt/SLO/GA471/2019/G8P[14]**	Cha	8	14	2	2	2	2	3	2	6	2	3
RVA/Human-tc/USA/DS-1/1976/G2P[4]	Hu	2	4	2	2	2	2	2	2	2	2	2
RVA/Human-wt/MWI/1473/2001/G8P[4]	Hu	8	4	2	2	2	2	2	2	2	2	2
**RVA/Red deer-wt/SLO/JE282/2017/G10/P[15]**	Red	10	15	2	2	2	2	11	2	6	2	3
**RVA/Red deer-wt/SLO/JE295/2017/G6/P[14]**	Red	6	14	2	2	2	2	11	2	6	2	3
RVA/Human-wt/KEN/AK26/2011/G2P[4]	Hu	2	4	2	2	2	2	2	1	2	2	2
RVA/Human-wt/JPN/KF17/2010/G6P[9]	Hu	6	9	2	2	2	2	3	2	3	3	3
RVA/Rabbit-wt/CHN/N5/1992/G3P[14]	Rab	3	14	17	3	3	3	9	1	1	3	2
RVA/Human-wt/AUS/V585/2011/G10P[14]	Hu	10	14	2	2	2	2	11	2	6	2	3
RVA/Human-wt/BRB/2012821133/2012/G4P[14]	Hu	4	14	1	1	1	1	8	1	1	1	1
RVA/Pig-tc/KOR/174–1/2006/G8P[7]	Pi	8	7	15	1	1	2	1	1	1	1	1
RVA/Human-wt/AUS/RCH272/2012/G3P[14]	Hu	3	14	2	3	3	3	9	2	6	2	3
RVA/Human-wt/USA/2012841174/2012/G8P[14]	Hu	8	14	2	3	2	2	3	2	6	2	3
RVA/Human-wt/USA/Wa/1974/G1P[8]	Hu	1	8	1	1	1	1	1	1	1	1	1
RVA/Human-wt/ITA/PR1300/2004/G8P[14]	Hu	8	14	2	2	2	2	3	2	6	2	3
RVA/Human-wt/ITA/PR1973/2009/G8P[14]	Hu	8	14	2	2	2	2	3	2	6	2	3
RVA/Human-tc/EGY/AS970/2012/G8P[14]	Hu	8	14	2	2	2	2	11	2	6	2	3
RVA/Human-wt/HUN/182–02/2002/G8P[14]	Hu	8	14	2	2	2	2	11	2	6	2	3
RVA/Roe deer-wt/SLO/D38-14/2014/G6P[15]	Roe	6	15	2	2	2	2	3	2	6	2	3
RVA/Roe deer-wt/SLO/D110-15/2015/G8P[14]	Roe	8	14	2	2	2	2	3	2	6	2	3
RVA/Human-wt/THA/SKT-27/2012/G6P[14]	Hu	6	14	2	2	2	2	3	2	6	2	3
RVA/Bovine/Northern Ireland/R1WTA17/2013/G6P[11]	Bo	6	11	2	2	2	2	3	2	6	2	3
RVA/Ovine/Northern Ireland/R2WTA65/2014/G10P[15]	Ov	10	15	2	2	2	2	11	2	6	2	3
**RVA/Roe deer-wt/SLO/SR100/2017/G6P[14]**	Roe	6	14	2	2	2	2	11	2	6	2	3
**RVA/Roe deer-wt/SLO/SR294/2017/G10P[15]**	Roe	10	15	2	2	2	2	3	2	6	2	3
**RVA/Roe deer-wt/SLO/SR333/2017/G10P[15]**	Roe	10	15	2	2	2	2	3	2	6	2	3
**RVA/Roe deer-wt/SLO/SR338/2017/G10P[15]**	Roe	10	15	2	2	2	2	3	2	6	2	3

## 3 Results

**Figure 1:**
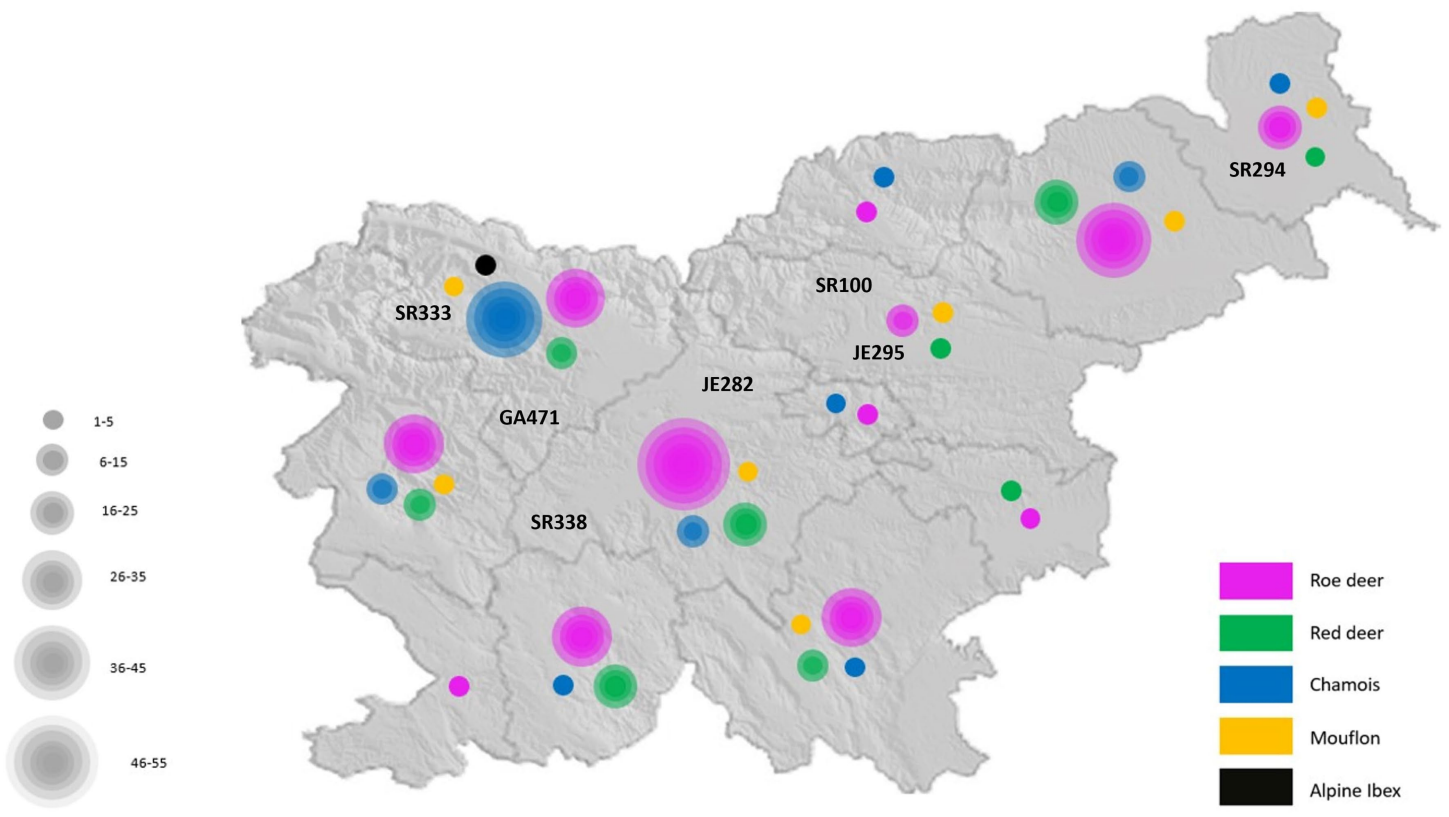
The geographical location of all sampled wild ruminants in Slovenia.

Four hundred and thirty-two fecal samples from different wild ruminants from across Slovenia (Figure 1) were tested for RVA. Seven of these samples were positive for RVA with real time RT-PCR, detecting the VP2 segment, which is known to detect a broad spectrum of RVA. Positive samples were sent for NGS sequencing and further bioinformatic and phylogenetic analyses were performed. The prevalence of RVA in wild ruminants in Slovenia, Europe between 2017 and 2021 was 2.15% in roe deer and less than 2% in red deer and chamois (Table 2). RVA-positive animals were found at different locations in Slovenia, of different ages and of both sexes (Figure 1; Table 3).

**Table 2:** Number (%) RVA positive animals among different species.

	Roe deer	Red deer	Chamois	Mouflon	Alpine ibex
Number all	249	93	78	10	2
Number (%) RVA positive	4 (1.61%)	2 (2.15%)	1 (1.28%)	0	0

**Table 3:** Age, sex, and location of RVA positive animals.

RVA Strain	Species	Age	Sex	Location
GA471	Chamois	Kid	Male	Poljane
JE282	Red deer	2 years	Female	Lukovica
JE295	Red deer	2 years	Female	Žalec
SR100	Roe deer	1 year	Male	Solčava
SR294	Roe deer	5 years	Male	Murska Sobota
SR333	Roe deer	2 years	Male	Kranjska Gora
SR338	Roe deer	1 year	Female	Medvedje Brdo

### 3.1 NGS analysis and complete genome construction

Seven complete RVA genome sequences of all 11 segments were generated and deposited in the GenBank under the accession numbers listed in Supplementary Table S1. The number of raw reads per sample ranged from 22 to 41 million (Supplementary Table S16). The average coverage of genome segments varied from 12.78 to 39865.36.

### 3.2 Rotavirus genome constellation

The Subspecies Classification for Rotavirus A tool analysis revealed that 7 RVA strains from this study have 4 different genome constellations (Table 1) and all 4 have a DS1-like backbone. Based on the whole genome constellation, there were four different genotypes circulating among wild ruminants in Slovenia between 2017 and 2021: G8-P[14]-I2-R2-C2-M2-A3-N2-T6-E2-H3, G6-P[14]-I2-R2-C2-M2-A11-N2-T6-E2-H3, G10-P[15]-I2-R2-C2-M2-A3-N2-T6-E2-H3 and G10-P[15]-I2-R2-C2-M2-A11-N2-T6-E2-H3.

### 3.3 Phylogenetic analysis of the genome segments

The genome constellations of the 42 RVA genomes used for phylogenetic analysis are listed in Table 1.

**Figure 2:**
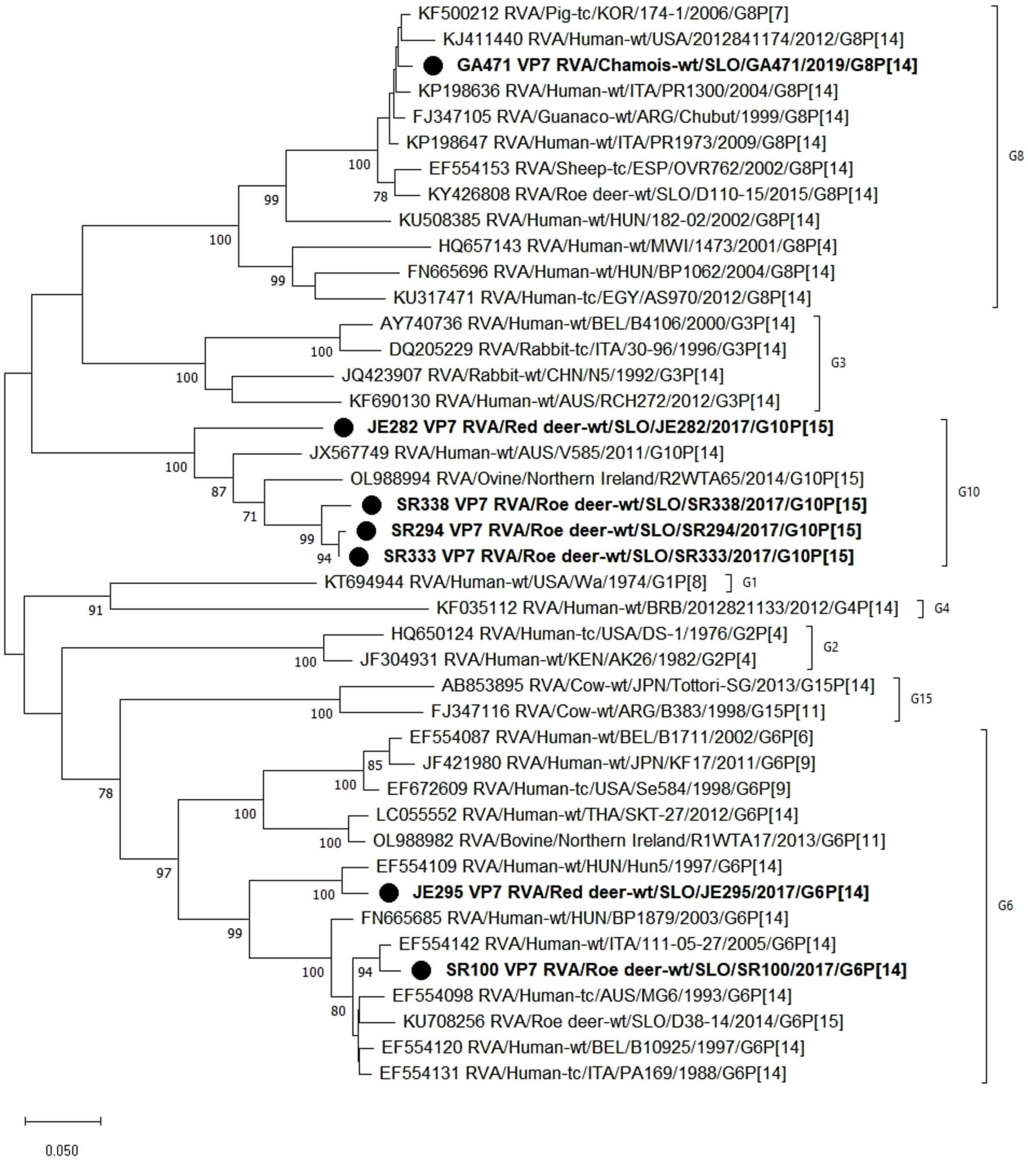
The Maximum likelihood phylogenetic tree on VP7 segment. Bootstrap values lower than 70 are not shown. The Slovenian wild ruminants’ strains are marked with circle.

Phylogenetic analysis of the VP4 segment shows that the RVA strains from wild ruminants from this study have 2 different P genotypes, the P[14] and P[15]. Strains with the P[15] genotype (JE282, SR294, SR333, and SR338) are most closely related to the previously found RVA genome of the Slovenian roe deer strain D38-14, with nucleotide identities varying between 88.5 and 99.4%. Other RVA strains from this study with P[14] genotype (GA471, JE295, and SR100) show a nucleotide identity of 97.4–98.5% with the Hungarian 182–02 and the Italian 111–05-27 human RVA strains (Figure 2).

**Figure 3:**
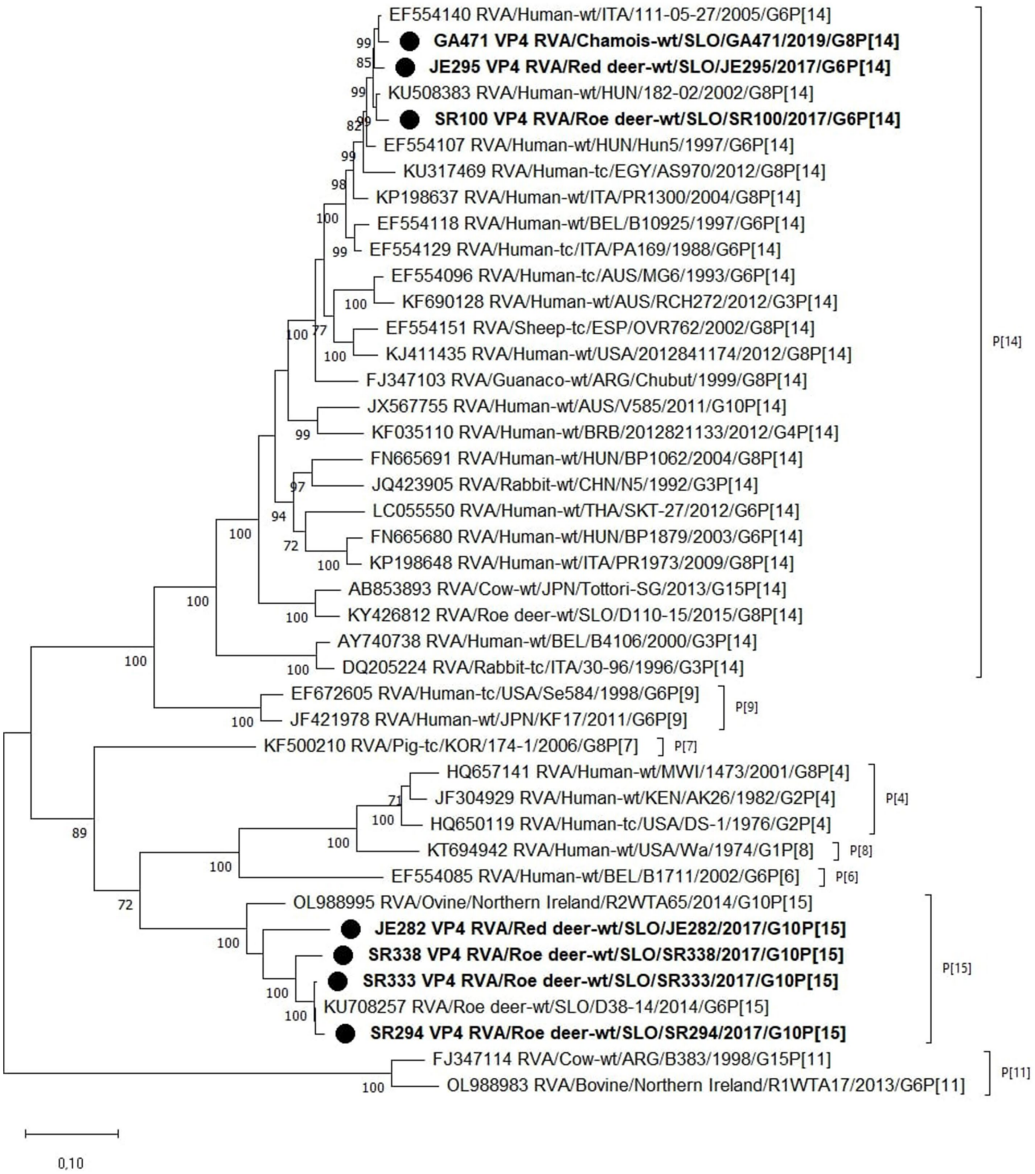
The Maximum likelihood phylogenetic tree on VP4 segment. Bootstrap values lower than 70 are not shown. The Slovenian wild ruminants’ strains are marked with circle.

Based on the phylogenetic analysis of the VP7 segment, 3 different G genotypes were found in the wild ruminants from this study, namely G6 (JE295 and SR100), G8 (GA471) and G10 (JE282, SR294, SR333, SR338). Strain JE295 with genotype G6 is most closely related to the Hungarian Hun5 human strain with 96.5% nucleotide identity. Another strain with G6 genotype is SR100, which is most similar to the Italian strain 111–05-27 with 97.8% nucleotide identity. The RVA strain GA471 with genotype G8 is most closely related to the porcine RVA strain 174–1 with 98.2% nucleotide identity and almost as closely related to the human RVA strain PR1973 with 98.1% nucleotide identity. Three strains with genotype G10 (SR294, SR333, SR338) are most similar to each other with a nucleotide identity of 96.6–99.4%, while strain JE282, also with genotype G10, is most similar to human RVA strain V585 with 86.4% nucleotide identity (Figure 3).

We also found different A genotypes (NSP1 segment); strains GA471, SR294, SR333, and SR338 have genotype A3 like the other roe deer RVAs D11-15 and D38-14. 3 RVA strains (JE282, JE295 and SR100) have genotype A11, like some human and sheep strains (Supplementary Figure S5). Regarding other genome segments, NSP2-5 (Supplementary Figures S6–S9), VP1-3 (Supplementary Figures S2–S4) and VP6 (Supplementary Figure S1), phylogenetic analysis showed that the wild ruminant RVA strains from this study have the same genotype with nucleotide identities between 48.9 and 99.6%. These results are shown in Supplementary Tables S9–S15.

## 4 Discussion

In this study, rotaviruses were investigated in wild ruminants in Slovenia, Europe from 2017 to 2021. We confirmed RVA for the first time in red deer and chamois. We found that the detection rate of RVA in roe deer was 1.61%, which corresponds to the result of the study from Germany (26). The detection rate of RVA in red deer was 2.15% and in chamois 1.28%. However, these results could not be compared with other studies as this was the first time that detection of RVA was reported in these species. Nevertheless, the detection rates in wild ruminants were still relatively low compared to domestic animals, possibly due to less contact with humans (27).

Two new G and [P] genotype combinations were found in wild ruminants (G6P[14] and G10P[15]) and one combination identical to a previously reported strain from Slovenia (G8P[14]) (18). All seven strains from this study had the same DS-1 like non-G/P genotype constellation (I2-R2-C2-M2-(A3/11)-N2-T6-E2-H3) of bovine and bovine-like strains, which is commonly found in RVA strains from artiodactyls such as cattle (15).

The G8P[14] and G6P[14] RVA genotypes with DS1-like genetic backbone are commonly found in animal species of the order *Artiodactyla* and are also known to cause human infections (12, 28–33). The RVA genotype G8P[14] has been detected sporadically in cattle, sheep, guanaco, vicuna, roe deer and in humans (12, 30–38). In this study, the RVA strain GA471 with the genotype G8P[14] was found in chamois. It showed high nucleotide identity with various human RVA strains in the VP4, VP2, VP6 and NSP3 segments, while in other segments it was more closely related to the RVA strains of roe deer, red deer and pigs. This strain also belonged to the DS-1-like group and had the same genome constellation G8-P[14]-I2-R2-C2-M2-A3-N2-T6-E2-H3 as the previously found Slovenian RVA strain D110-15, whose host was roe deer (38).

The RVA genotype G6P[14] has been detected in some animal species, such as antelope, goat, cattle and also in humans (28, 29, 34, 39–43). In the present study, the RVA G6P[14] strain was detected for the first time in red deer and roe deer species. The RVA strain JE295 was found in red deer and was most closely related to human RVA strains according to whole genome phylogenetic analysis in all segments except VP2, VP3 and NSP1, in which it was most closely related to red deer and chamois RVA strain. Similarly, RVA strain SR100 found in roe deer had the highest nucleotide identity with human RVA strains except in segments VP6, VP3 and NSP1, in which the closest relatives were RVA strains isolated from red deer and sheep. Both strains, JE295 and SR100, had the same genotype constellation G6P[14] I2-R2-C2-M2-A11-N2-T6-E2-H3, which had also been found in humans, more specifically in children with diarrheal diseases in several countries (40–42). RVA strains with G6P[14] found in other studies (40–42) had similar characteristics to those from Slovenia.

Matthijnssens et al. (34) suggest that human P[14] (G6 and G8) rotaviruses may have originated in a ruminant (cattle, goat, sheep or antelope) or camelid (guanaco), based on the conserved overall genetic constellation found in a large number of these animals. In this respect, sheep, goats or cattle are probably the main reservoir of P[14] rotaviruses for humans worldwide, as these animals live close to humans. Since rotaviruses are frequently transmitted among members of the order *Artiodactyla*, human P[14] rotaviruses probably do not have a single common animal source. Based on the evolutionary relationships observed in the study by Matthijnssens et al. (34) and later studies reporting P[14] strains (30, 32, 35–37, 43, 44), these strains are complex and likely involve multiple animal-to-animal transmissions, reassortments, and subsequent transmissions from an animal to a human. Similar results were also obtained in our study, supporting this hypothesis.

Another G-P combination of RVA was found in this study, G10P[15], which was detected in 4 samples from red deer and roe deer (RVA strains JE282, SR294, SR333 and SR338). The G10P[15] genotypes were predominantly bovine strains, but had also been found in sheep and camels (3, 45–48). The RVA strain JE282 detected in red deer was found to be most closely related to roe deer, red deer and chamois RVA strains in most segments, and to the human RVA strains in segments VP7, VP6, NSP4, and NSP5. Another RVA strain SR294, which was found in roe deer, showed the greatest similarities in the nucleotide sequence in most segments with roe deer, bovine and porcine RVA strains, and only in two segments (NSP2 and NSP4) with human RVA strains. The RVA strains SR333 and SR338, both isolated from roe deer, showed the greatest similarities in nucleotide sequence to RVA strains found in animals of the order *Artiodactyla*. Although there have been no reports of the G10P[15] genotype in humans, other P combinations with G10 have been found in humans (49, 50). However, the genome constellation of the roe deer RVA strains (SR294, SR333, and SR338) differed in the non-structural NSP1 segment from that of the red deer strain (JE282). The red deer strain had genotype A11, whereas the roe deer strains had genotype A3. The phylogenetic tree of the NSP1 segment in this study showed two different A genotypes among wild ruminants, A3 and A11, which were detected in cows (51), roe deer (38) and humans (34, 40). It is not known what effect the differences between the A genotypes have on the host or virus transmission (52).

## 5 Conclusion

In this study, new genotype combinations (G6P[14] and G10P[15]) were discovered alongside a known one (G8P[14, 12]) in wild ruminants in Slovenia from 2017 to 2021. G6P[14] was found in red deer and roe deer, G8P[14] in chamois and G10P[15] in red deer and roe deer. The genotypes G6P[14] and G8P[14] showed zoonotic potential as they have similarities with human RVA strains. On the other hand, different RVA genotypes can infect different hosts and possibly cross species, so the question of the zoonotic origin of RVA remains open. Currently, there is insufficient evidence to determine whether humans or animals are the primary reservoir for RVA. Further research is needed to decipher the transmission dynamics.

## Data availability statement

The datasets presented in this study can be found in online repositories. The names of the repository/repositories and accession number(s) can be found in the article/Supplementary material.

## Ethics statement

Ethical approval was not required for the study involving animals in accordance with the local legislation and institutional requirements because all samples were collected at postmortem.

## Author contributions

PŠ: Data curation, Formal analysis, Investigation, Methodology, Project administration, Software, Validation, Visualization, Writing – original draft. DŽ: Methodology, Resources, Writing – review & editing. GV: Methodology, Resources, Writing – review & editing. UK: Conceptualization, Funding acquisition, Investigation, Methodology, Resources, Supervision, Writing – original draft.

## References

[1] Kumar D Shepherd FK Springer NL Mwangi W Marthaler DG. Rotavirus infection in swine: genotypic diversity, immune responses, and role of gut microbiome in rotavirus immunity. Pathogens. (2022) 11:1078. doi: 10.3390/pathogens11101078 36297136 PMC9607047

[2] Papp H László B Jakab F Ganesh B De Grazia S Matthijnssens J. Review of group A rotavirus strains reported in swine and cattle. Vet Microbiol. (2013) 165:190–9. doi: 10.1016/j.vetmic.2013.03.020 23642647 PMC7117210

[3] Papp H Malik YS Farkas SL Jakab F Martella V Bányai K. Rotavirus strains in neglected animal species including lambs, goats and camelids. Virusdisease. (2014) 25:215–22. doi: 10.1007/s13337-014-0203-2, PMID: 25674588 PMC4188177

[4] Bailey KE Gilkerson JR Browning GF. Equine rotaviruses-current understanding and continuing challenges. Vet Microbiol. (2013) 167:135–44. doi: 10.1016/j.vetmic.2013.07.010 23932076 PMC7117381

[5] Dhama K Chauhan RS Mahendran M Malik SVS. Rotavirus diarrhea in bovines and other domestic animals. Vet Res Commun. (2009) 33:1–23. doi: 10.1007/s11259-008-9070-x, PMID: 18622713 PMC7088678

[6] Dennehy PH. Transmission of rotavirus and other enteric pathogens in the home. Pediatr Infect Dis J. (2000) 19:S103–5. doi: 10.1097/00006454-200010001-00003, PMID: 11052397

[7] Wilhelm B Waddell L Greig J Rajić A Houde A McEwen SA. A scoping review of the evidence for public health risks of three emerging potentially zoonotic viruses: hepatitis E virus, norovirus, and rotavirus. Prev Vet Med. (2015) 119:61–79. doi: 10.1016/j.prevetmed.2015.01.015, PMID: 25681862

[8] Matthijnssens J Ciarlet M McDonald SM Attoui H Bányai K Brister JR. Uniformity of rotavirus strain nomenclature proposed by the rotavirus classification working group (RCWG). Arch Virol. (2011) 156:1397–413. doi: 10.1007/s00705-011-1006-z, PMID: 21597953 PMC3398998

[9] Pesavento JB Crawford SE Estes MK Venkataram Prasad BV. Rotavirus proteins: structure and assembly. Curr Top Microbiol Immunol. (2006) 309:189–219. doi: 10.1007/3-540-30773-7_7, PMID: 16913048

[10] Knipe DM Howley PM Cohen JI Griffin DE Lamb RA Martin MA. Fields virology. Philadelphia, PA: Lippincott Williams & Wilkins (2013).

[11] RCWG. List of accepted genotypes. Laboratory of Viral Metagenomics (2023). Available at: https://rega.kuleuven.be/cev/viralmetagenomics/virus-classification/rcwg

[12] Bányai K Papp H Dandár E Molnár P Mihály I Van Ranst M. Whole genome sequencing and phylogenetic analysis of a zoonotic human G8P[14] rotavirus strain. Infect Genet Evol. (2010) 10:1140–4. doi: 10.1016/j.meegid.2010.05.001, PMID: 20471499

[13] Malik YS Bhat S Dar PS Sircar S Dhama K Singh RK. Evolving rotaviruses, interspecies transmission and Zoonoses. Open Virol J. (2020) 14:1–6. doi: 10.2174/1874357902014010001

[14] Chamsai E Charoenkul K Udom K Jairak W Chaiyawong S Amonsin A. Genetic characterization and evidence for multiple reassortments of rotavirus a G3P[3] in dogs and cats in Thailand. Front Vet Sci. (2024) 11:1415771. doi: 10.3389/fvets.2024.1415771, PMID: 38855413 PMC11157116

[15] Matthijnssens J Ciarlet M Heiman E Arijs I Delbeke T McDonald SM. Full genome-based classification of rotaviruses reveals a common origin between human Wa-like and porcine rotavirus strains and human DS-1-like and bovine rotavirus strains. J Virol. (2008) 82:3204–19. doi: 10.1128/JVI.02257-07, PMID: 18216098 PMC2268446

[16] Matthijnssens J Van Ranst M. Genotype constellation and evolution of group a rotaviruses infecting humans. Curr Opin Virol. (2012) 2:426–33. doi: 10.1016/j.coviro.2012.04.007, PMID: 22683209

[17] Martella V Bányai K Matthijnssens J Buonavoglia C Ciarlet M. Zoonotic aspects of rotaviruses. Vet Microbiol. (2010) 140:246–55. doi: 10.1016/j.vetmic.2009.08.028, PMID: 19781872

[18] Gutiérrez-Aguirre I Steyer A Boben J Gruden K Poljšak-Prijatelj M Ravnikar M. Sensitive detection of multiple rotavirus genotypes with a single reverse transcription-real-time quantitative PCR assay. J Clin Microbiol. (2008) 46:2547–54. doi: 10.1128/JCM.02428-07, PMID: 18524966 PMC2519481

[19] Zhao J Ohsumi TK Kung JT Ogawa Y Grau DJ Sarma K. Genome-wide identification of polycomb-associated RNAs by RIP-seq. Mol Cell. (2010) 40:939–53. doi: 10.1016/j.molcel.2010.12.011, PMID: 21172659 PMC3021903

[20] Nurk S Meleshko D Korobeynikov A Pevzner PA. MetaSPAdes: a new versatile metagenomic assembler. Genome Res. (2017) 27:824–34. doi: 10.1101/gr.213959.116, PMID: 28298430 PMC5411777

[21] Buchfink B Xie C Huson DH. Fast and sensitive protein alignment using DIAMOND. Nat Methods. (2015) 12:59–60. doi: 10.1038/nmeth.3176, PMID: 25402007

[22] Huson DH Beier S Flade I Górska A El-Hadidi M Mitra S. MEGAN Community edition - interactive exploration and analysis of large-scale microbiome sequencing data. PLoS Comput Biol. (2016) 12:e1004957. doi: 10.1371/journal.pcbi.1004957, PMID: 27327495 PMC4915700

[23] Subspecies Classification Service. BV-BRC (2023). Available at: https://www.bv-brc.org/app/SubspeciesClassification

[24] Katoh K Standley DM. MAFFT multiple sequence alignment software version 7: improvements in performance and usability. Mol Biol Evol. (2013) 30:772–80. doi: 10.1093/molbev/mst010, PMID: 23329690 PMC3603318

[25] Tamura K Stecher G Kumar S. MEGA11: molecular evolutionary genetics analysis version 11. Mol Biol Evol. (2021) 38:3022–7. doi: 10.1093/molbev/msab120, PMID: 33892491 PMC8233496

[26] Althof N Trojnar E Johne R. Rotaviruses in wild ungulates from Germany, 2019–2022. Microorganisms. (2023) 11:566. doi: 10.3390/microorganisms11030566, PMID: 36985140 PMC10058221

[27] Díaz Alarcón RG Liotta DJ Miño S. Zoonotic RVA: state of the art and distribution in the animal world. Viruses. (2022) 14:2554. doi: 10.3390/v14112554, PMID: 36423163 PMC9694813

[28] Matthijnssens J Bilcke J Ciarlet M Martella V Bányai K Rahman M. Rotavirus disease and vaccination: impact on genotype diversity. Future Microbiol. (2009) 4:1303–16. doi: 10.2217/fmb.09.96 19995190

[29] Mullick S Mukherjee A Ghosh S Pazhani GP Sur D Manna B. Genomic analysis of human rotavirus strains G6P[14] and G11P[25] isolated from Kolkata in 2009 reveals interspecies transmission and complex reassortment events. Infect Genet Evol. (2013) 14:15–21. doi: 10.1016/j.meegid.2012.11.010, PMID: 23219735

[30] Delogu R Ianiro G Morea A Chironna M Fiore L Ruggeri FM. Molecular characterization of two rare human G8P[14] rotavirus strains, detected in Italy in 2012. Infect Genet Evol. (2016) 44:303–12. doi: 10.1016/j.meegid.2016.07.018, PMID: 27449953

[31] Alaoui Amine S Melloul M El Alaoui MA Touil N El Fahime E. Full-length genome analysis of the first human G8P[14] rotavirus strain from Morocco suggests evidence of zoonotic transmission. Virus Genes. (2019) 55:465–78. doi: 10.1007/s11262-019-01677-9, PMID: 31197545

[32] Gautam R Mijatovic-Rustempasic S Roy S Esona MD Lopez B Mencos Y. Full genomic characterization and phylogenetic analysis of a zoonotic human G8P[14] rotavirus strain detected in a sample from Guatemala. Infect Genet Evol. (2015) 33:206–11. doi: 10.1016/j.meegid.2015.05.004, PMID: 25952569 PMC5803796

[33] Wu F-T Báanyai K Wu HS Yang DCF Lin JS Hsiung CA. Identification of a G8P[14] rotavirus isolate obtained from a Taiwanese child: evidence for a relationship with bovine rotaviruses. Jpn J Infect Dis. (2012) 65:455–7. doi: 10.7883/yoken.65.455, PMID: 22996226 PMC8211372

[34] Matthijnssens J Potgieter CA Ciarlet M Parreño V Martella V Bányai K. Are human P[14] rotavirus strains the result of interspecies transmissions from sheep or other ungulates that belong to the mammalian order Artiodactyla? J Virol. (2009) 83:2917–29. doi: 10.1128/JVI.02246-08, PMID: 19153225 PMC2655590

[35] Steyer A Naglič T Jamnikar-Ciglenečki U Kuhar U. Detection and whole-genome analysis of a zoonotic G8P[14] rotavirus strain isolated from a child with diarrhea. Genome Announc. (2017) 5:e01053-17. doi: 10.1128/genomeA.01053-17, PMID: 29025940 PMC5637500

[36] Marton S Dóró R Fehér E Forró B Ihász K Varga-Kugler R. Whole genome sequencing of a rare rotavirus from archived stool sample demonstrates independent zoonotic origin of human G8P[14] strains in Hungary. Virus Res. (2017) 227:96–103. doi: 10.1016/j.virusres.2016.09.012 27671785

[37] Medici MC Tummolo F Bonica MB Heylen E Zeller M Calderaro A. Genetic diversity in three bovine-like human G8P[14] and G10P[14] rotaviruses suggests independent interspecies transmission events. J Gen Virol. (2015) 96:1161–8. doi: 10.1099/vir.0.000055, PMID: 25614586

[38] Jamnikar-Ciglenecki U Kuhar U Steyer A Kirbis A. Whole genome sequence and a phylogenetic analysis of the G8P[14] group a rotavirus strain from roe deer. BMC Vet Res. (2017) 13:353. doi: 10.1186/s12917-017-1280-4, PMID: 29178883 PMC5702219

[39] Sawant PM Digraskar S Gopalkrishna V. Molecular characterization of unusual G10P[33], G6P[14] genomic constellations of group a rotavirus and evidence of zooanthroponosis in bovines. Infect Genet Evol. (2020) 84:104385. doi: 10.1016/j.meegid.2020.104385 32522623

[40] El Sherif M Esona MD Wang Y Gentsch JR Jiang B Glass RI. Detection of the first G6P[14] human rotavirus strain from a child with diarrhea in Egypt. Infect Genet Evol. (2011) 11:1436–42. doi: 10.1016/j.meegid.2011.05.012, PMID: 21640199

[41] Damanka S Lartey B Agbemabiese C Dennis FE Adiku T Nyarko K. Detection of the first G6P[14] human rotavirus strain in an infant with diarrhoea in Ghana. Virol J. (2016) 13:1–7. doi: 10.1186/s12985-016-0643-y 27832798 PMC5103419

[42] Damanka SA Dennis FE Lartey BL Nyarko KM Agbemabiese CA Armah GE. Next-generation sequencing of a human-animal reassortant G6P[14] rotavirus a strain from a child hospitalized with diarrhoea. Arch Virol. (2020) 165:1003–5. doi: 10.1007/s00705-020-04543-4, PMID: 32037490

[43] Bányai K Martella V Molnár P Mihály I Van Ranst M Matthijnssens J. Genetic heterogeneity in human G6P[14] rotavirus strains detected in Hungary suggests independent zoonotic origin. J Infect. (2009) 59:213–5. doi: 10.1016/j.jinf.2009.06.009, PMID: 19608281

[44] Cowley D Donato CM Roczo-Farkas S Kirkwood CD. Novel G10P[14] rotavirus strain, Northern Territory, Australia. Emerg Infect Dis. (2013) 19:1324–7. doi: 10.3201/eid1908.121653, PMID: 23876354 PMC3739504

[45] Rajendran P Kang G. Molecular epidemiology of rotavirus in children and animals and characterization of an unusual G10P[15] strain associated with bovine diarrhea in South India. Vaccine. (2014) 32:A89–94. doi: 10.1016/j.vaccine.2014.03.026 25091687

[46] Gazal S Mir IA Iqbal A Taku AK Kumar B Bhat MA. Ovine rotaviruses. Open Vet J. (2011) 5:50–4485. doi: 10.5455/OVJ.2011.v1.i0.p50 PMC4655755 26623281

[47] Papp H Al-Mutairi L Chehadeh W Farkas S Lengyel G Jakab F. Novel NSP4 genotype in a camel G10P[15] rotavirus strain. Acta Microbiol Immunol Hung. (2012) 59:411–21. doi: 10.1556/amicr.59.2012.3.11, PMID: 22982644

[48] Chen Y Zhu W Sui S Yin Y Hu S Zhang X. Whole genome sequencing of lamb rotavirus and comparative analysis with other mammalian rotaviruses. Virus Genes. (2009) 38:302–10. doi: 10.1007/s11262-009-0332-7, PMID: 19214729

[49] Armah GE Hoshino Y Santos N Binka F Damanka S Adjei R. The global spread of rotavirus G10 strains: detection in Ghanaian children hospitalized with Diarrhoea. J Infect Dis. (2010) 202:S231–8. doi: 10.1086/653572, PMID: 20684709 PMC2954461

[50] Ghosh S Alam MM Ahmed MU Talukdar RI Paul SK Kobayashi N. Complete genome constellation of a caprine group a rotavirus strain reveals common evolution with ruminant and human rotavirus strains. J Gen Virol. (2010) 91:2367–73. doi: 10.1099/vir.0.022244-0 20505013

[51] Masuda T Nagai M Yamasato H Tsuchiaka S Okazaki S Katayama Y. Identification of novel bovine group a rotavirus G15P[14] strain from epizootic diarrhea of adult cows by de novo sequencing using a next-generation sequencer. Vet Microbiol. (2014) 171:66–73. doi: 10.1016/j.vetmic.2014.03.009, PMID: 24725447 PMC7127257

[52] Hou G Zeng Q Matthijnssens J Greenberg HB Ding S. Rotavirus NSP1 contributes to intestinal viral replication, pathogenesis, and transmission. mBio. (2021) 12:e0320821. doi: 10.1128/mBio.03208-21, PMID: 34903043 PMC8669464

