# Supplementary material for: Genomic revelations: investigating rotavirus a presence in wild ruminants and its zoonotic potential
Source: Front Vet Sci. 2024 Aug 15;11:1429654. doi: 10.3389/fvets.2024.1429654 (PMC11358691; doi:10.3389/fvets.2024.1429654)
Supplement: Supplementary file 1 [file Data_Sheet_1.pdf]

Supplementary Material

1 Supplementary Figures and Tables

Supplementary Table 1. Accession numbers of 11 segments of RVA whole genomes

Name	Segment	Accession number
GA471_NSP1	5	OR270737
GA471_NSP2	8	OR270738
GA471_NSP3	7	OR270739
GA471_NSP4	10	OR270740
GA471_NSP5_NSP6	11	OR270741
GA471_VP1	1	OR270742
GA471_VP2	2	OR270743
GA471_VP3	3	OR270744
GA471_VP4	4	OR270745
GA471_VP6	6	OR270746
GA471_VP7	9	OR270747
JE282_NSP1	5	OR270748
JE282_NSP2	8	OR270749
JE282_NSP3	7	OR270750
JE282_NSP4	10	OR270751
JE282_NSP5_NSP6	11	OR270752
JE282_VP1	1	OR270753
JE282_VP2	2	OR270754
JE282_VP3	3	OR270755
JE282_VP4	4	OR270756
JE282_VP6	6	OR270757
JE282_VP7	9	OR270758
JE295_NSP1	5	OR270759
JE295_NSP2	8	OR270760
JE295_NSP3	7	OR270761
JE295_NSP4	10	OR270762
JE295_NSP5_NSP6	11	OR270763
JE295_VP1	1	OR270764
JE295_VP2	2	OR270765
JE295_VP3	3	OR270766
JE295_VP4	4	OR270767

JE295_VP6	6	OR270768
JE295_VP7	9	OR270769
SR100_NSP1	5	OR270770
SR100_NSP2	8	OR270771
SR100_NSP3	7	OR270772
SR100_NSP4	10	OR270773
SR100_NSP5_NSP6	11	OR270774
SR100_VP1	1	OR270775
SR100_VP2	2	OR270776
SR100_VP3	3	OR270777
SR100_VP4	4	OR270778
SR100_VP6	6	OR270779
SR100_VP7	9	OR270780
SR294_NSP1	5	OR270781
SR294_NSP2	8	OR270782
SR294_NSP3	7	OR270783
SR294_NSP4	10	OR270784
SR294_NSP5_NSP6	11	OR270785
SR294_VP1	1	OR270786
SR294_VP2	2	OR270787
SR294_VP3	3	OR270788
SR294_VP4	4	OR270789
SR294_VP6	6	OR270790
SR294_VP7	9	OR270791
SR333_NSP1	5	OR270792
SR333_NSP2	8	OR270793
SR333_NSP3	7	OR270794
SR333_NSP4	10	OR270795
SR333_NSP5_NSP6	11	OR270796
SR333_VP1	1	OR270797
SR333_VP2	2	OR270798
SR333_VP3	3	OR270799
SR333_VP4	4	OR270800
SR333_VP6	6	OR270801
SR333_VP7	9	OR270802
SR338_NSP1	5	OR270803
SR338_NSP2	8	OR270804
SR338_NSP3	7	OR270805

SR338_NSP4	10	OR270806
SR338_NSP5_NSP6	11	OR270807
SR338_VP1	1	OR270808
SR338_VP2	2	OR270809
SR338_VP3	3	OR270810
SR338_VP4	4	OR270811
SR338_VP6	6	OR270812
SR338_VP7	9	OR270813

Supplementary Table 2. Reference genomes from GenBank based on the blastn search of contigs for GA471 strain.

GA471 Gene	Genotype	Strain	Accession number	Identity	Host
VP7	G8	88977	KJ940165	98.7 %	Canine
VP4	P14	111-05-27	EF554140	98.5 %	Human
VP6	I2	RRVA/L232	MN626422	98.5 %	Rabbit
VP1	R2	182-02	KU508380	97.1 %	Human
VP2	C2	DB2015-066	LC367315	97.6 %	Human
VP3	M2	MAR/S19	MN067446	96.0 %	Goat
NSP1	A3	NCDV	HQ186290	97.0 %	Bovine
NSP2	N2	BP1879	FN665683	97.1 %	Human
NSP3	T6	B10925	EF554123	98.6 %	Human
NSP4	E2	20111204	OM283126	97.7 %	Human
NSP5	H2	R1WTA12	OL988956	98.7 %	Bovine

Supplementary Table 3. Reference genomes from GenBank based on the blastn search of contigs for JE282 strain.

JE282 Gene	Genotype	Strain	Accession number	Identity	Host
VP7	G10	E29TR	FJ598311	96.1 %	Bovine
VP4	P15	LLR	JQ013506	89.1%	Ovine
VP6	I2	182-02	KU508384	96.1 %	Human
VP1	R2	PR1300	KP198639	98.4 %	Human
VP2	C2	Hun5	EF554105	96.7 %	Human
VP3	M2	MAR/S19	MN067446	96.0 %	Goat
NSP1	A11	BP1879	FN665681	93.8 %	Human
NSP2	N2	BP1879	FN665683	97.6 %	Human

NSP3	T6	B10925	EF554123	98.5 %	Human
NSP4	E2	20111204	OM283126	97.9 %	Human
NSP5	H2	R1WTA12	OL988956	98.7 %	Bovine

Supplementary Table 4. Reference genomes from GenBank based on the blastn search of contigs for JE295 strain.

JE295 Gene	Genotype	Strain	Accession number	Identity	Host
VP7	G6	Hun5	EF554109	96.5 %	Human
VP4	P14	111-05-27	EF554140	98.1 %	Human
VP6	I2	Nov10-N397	HQ611011	98.2 %	Human
VP1	R2	PR1300	KP198639	98.1 %	Human
VP2	C2	Hun5	EF554105	97.2 %	Human
VP3	M2	MAR/S19	MN067446	95.9 %	Goat
NSP1	A11	BP1879	FN665681	94.3 %	Human
NSP2	N2	BP1879	FN665683	98.0 %	Human
NSP3	T6	B10925	EF554123	98.9 %	Human
NSP4	E2	BP1879	FN665686	94.7 %	Human
NSP5	H2	GER29-14	KX880446	99.0 %	Human

Supplementary Table 5. Reference genomes from GenBank based on the blastn search of contigs for SR100 strain.

SR100 Gene	Genotype	Strain	Accession number	Identity	Host
VP7	G6	11/05/2027	EF554142	97.9 %	Human
VP4	P14	182-02	KU508383	98.1 %	Human
VP6	I2	OVR762	EF554152	98.1 %	Ovine
VP1	R2	PR1300	KP198639	98.3 %	Human
VP2	C2	Amasya-1	KX212873	97.2 %	Bovine
VP3	M2	MAR/S19	MN067446	96.6 %	Goat
NSP1	A11	BP1879	FN665681	93.4 %	Human
NSP2	N2	BP1879	FN665683	97.4 %	Human
NSP3	T6	Cat2	EU708964	95.5 %	Feline
NSP4	E2	182-02	KU508378	94.6 %	Human
NSP5	H2	Ghan-113	KP882636	98.2 %	Human

Supplementary Table 6. Reference genomes from GenBank based on the blastn search of contigs for SR294 strain.

SR294 Gene	Genotype	Strain	Accession number	Identity	Host
VP7	G10	KK3	LC133563	92.5 %	Bovine
VP4	P15	D38-14	KU708257	98.8 %	Roe Deer
VP6	I2	MVS-BRV4	KC215501	97.6 %	Vaccine
VP1	R2	A44	LC133569	96.0 %	Bovine
VP2	C2	BP1879	FN665678	98.2 %	Human
VP3	M2	GB-25	LC553619	96.7 %	Bovine-tc
NSP1	A3	D110-15	KY426803	96.2 %	Roe Deer
NSP2	N2	VEN/RRV_NB1215_31	LC438930	98.2 %	Human
NSP3	T6	D38-14	KU708264	99.0 %	Roe Deer
NSP4	E2	TURV01	JF327746	98.7 %	Turkey
NSP5	H2	VU12-13-42	MF168105	97.3 %	Human

Supplementary Table 7. Reference genomes from GenBank based on the blastn search of contigs for SR333 strain.

SR333 Gene	Genotype	Strain	Accession number	Identity	Host
VP7	G10	KK3	LC133563	92.5 %	Bovine
VP4	P15	D38-14	KU708257	99.4 %	Roe Deer
VP6	I2	MVS-BRV4	KC215501	97.8 %	Vaccine
VP1	R2	A44	LC133569	96.1 %	Bovine
VP2	C2	BP1879	FN665678	98.2 %	Human
VP3	M2	MAR/S19	MN067446	96.4 %	Goat
NSP1	A3	NCDV	HQ186290	96.8 %	Bovine
NSP2	N2	BP1879	FN665683	97.4 %	Human
NSP3	T6	BA01	KX024675	98.3 %	Human
NSP4	E2	20111204	OM283126	97.4 %	Human
NSP5	H2	GER29-14	KX880446	98.5 %	Human

Supplementary Table 8. Reference genomes from GenBank based on the blastn search of contigs for SR338 strain.

SR338 Gene	Genotype	Strain	Accession number	Identity	Host

VP7	G10	61A	LC133541	91.9 %	Bovine-tc
VP4	P15	D38-14	KU708257	99.4 %	Roe Deer
VP6	I2	Nov10-N397	HQ611011	98.4 %	Human
VP1	R2	UKg9P	GQ225785	95.1 %	Vaccine
VP2	C2	BP1879	FN665678	97.8 %	Human
VP3	M2	CP-1	FJ560906	98.1 %	Bovine
NSP1	A3	D110-15	KY426803	96.8 %	Roe Deer
NSP2	N2	61A	LC133543	96.8 %	Bovine-tc
NSP3	T6	Chubut	FJ347108	96.5 %	Guanaco
NSP4	E2	D38-14	KU708265	97.2 %	Roe Deer
NSP5	H2	VU12-13-42	MF168105	97.8 %	Human

Supplementary Table 9. Whole genome nucleotide comparison to representative strains from Genbank based on percents of nucleotide identity for strain GA471

Gene	Strain GA471		Strains in the Genbank with the closest nucleotide identity based on phylogenetic analysis			
	Genotype	Accession no.	Strain	Accession no.	Nt. Identity (%)	Host
VP7	G8	OR270747	174-1	KF500212	98,161	Pig
			PR1973	KP198647	98,059	Human
VP4	P[14]	OR270745	111-05-27	EF554140	98,541	Human
			182-02	KU508383	97,382	Human
VP6	I2	OR270746	PR1973	KP198649	98,24	Human
			111-05-27	EF554141	98,156	Human
VP1	R2	OR270742	JE282	OR270753	98,79	Red deer
			PR1300	KP198639	98,488	Human
VP2	C2	OR270743	B10925	EF554116	97,123	Human
			111-05-27	EF554138	97,048	Human
VP3	M2	OR270744	SR333	OR270799	99,481	Roe deer

			JE295	OR270766	98,684	Red deer
NSP1	A3	OR270737	SR333	OR270792	99,593	Roe deer
			SR338	OR270803	96,68	Roe deer
NSP2	N2	OR270738	SR333	OR270793	99,476	Roe deer
			PR1300	KP198643	98,426	Human
NSP3	T6	OR270739	B10925	EF554123	98,607	Human
			111-05-27	EF554145	98,499	Human
NSP4	E2	OR270740	SR333	OR270795	99,62	Roe deer
			PR1300	KP198645	97,913	Human
NSP5	H3	OR270741	SR333	OR270796	99,831	Roe deer
			PR1300	KP198646	99,494	Human

Supplementary Table 10. Whole genome nucleotide comparison to representative strains from Genbank based on percents of nucleotide identity for strain JE282

Gene	Strain JE282		Strains in the Genbank with the closest nucleotide identity based on phylogenetic analysis			
	Genotype	Accession no.	Strain	Accession no.	Nt. Identity (%)	Host
VP7	G10	OR270758	V585	JX567749	86,415	Human
			SR333	OR270802	86,159	Roe deer
VP4	P[15]	OR270756	SR333	OR270800	88,605	Roe deer
			D38-14	KU708257	88,541	Roe deer
VP6	I2	OR270757	182-02	KU508384	96,144	Human
			Hun5	EF554108	95,725	Human
VP1	R2	OR270753	GA471	OR270742	98,79	Chamois
			PR1300	KP198639	98,36	Human

VP2	C2	OR270754	JE295	OR270765	99,205	Red deer
			Hun5	EF554105	96,669	Human
VP3	M2	OR270755	GA471	OR270744	98,135	Chamois
			JE295	OR270766	98,095	Red deer
NSP1	A11	OR270748	JE295	OR270759	98,103	Red deer
			SR100	OR270770	96,07	Roe deer
NSP2	N2	OR270749	JE295	OR270760	99,057	Red deer
			PR1300	KP198643	99,056	Human
NSP3	T6	OR270750	JE295	OR270761	98,767	Red deer
			B10925	EF554123	98,553	Human
NSP4	E2	OR270751	PR1300	KP198645	97,913	Human
			GA471	OR270740	97,723	Chamois
NSP5	H3	OR270752	PR1300	KP198646	99,494	Human
			GA471	OR270741	99,325	Chamois

Supplementary Table 11. Whole genome nucleotide comparison to representative strains from Genbank based on percents of nucleotide identity for strain JE295

Gene	Strain JE295		Strains in the Genbank with the closest nucleotide identity based on phylogenetic analysis			
	Genotype	Accession no.	Strain	Accession no.	Nt. Identity (%)	Host
VP7	G6	OR270769	Hun5	EF554109	96,527	Human
			B10925	EF554120	87,436	Human
VP4	P[14]	OR270767	111-05-27	EF554140	98,112	Human
			182-02	KU508383	97,983	Human
VP6	I2	OR270768	PA169	EF554130	98,24	Human

			SR338	OR270812	97,318	Roe deer
VP1	R2	OR270764	PR1300	KP198639	98,074	Human
			JE282	OR270753	98,026	Red deer
VP2	C2	OR270765	JE282	OR270754	99,205	Red deer
			Hun5	EF554105	97,161	Human
VP3	M2	OR270766	GA471	OR270744	98,684	Chamois
			SR333	OR270799	98,484	Roe deer
NSP1	A11	OR270759	JE282	OR270748	98,103	Red deer
			SR100	OR270770	96,477	Roe deer
NSP2	N2	OR270760	PR1300	KP198643	99,161	Human
			JE282	OR270749	99,057	Red deer
NSP3	T6	OR270761	B10925	EF554123	98,928	Human
			111-05-27	EF554145	98,821	Human
NSP4	E2	OR270762	BP1879	FN665686	94,687	Human
			AS970	KU317464	92,6	Human
NSP5	H3	OR270763	PR1300	KP198646	99,494	Human
			JE282	OR270752	98,988	Red deer

Supplementary Table 12. Whole genome nucleotide comparison to representative strains from Genbank based on percents of nucleotide identity for strain SR100

Gene	Strain SR100		Strains in the Genbank with the closest nucleotide identity based on phylogenetic analysis			
	Genotype	Accession no.	Strain	Accession no.	Nt. Identity (%)	Host
VP7	G6	OR270780	111-05-27	EF554142	97,855	Human

			B10925	EF554120	96,323	Human
VP4	P[14]	OR270778	182-02	KU508383	98,069	Human
			111-05-27	EF554140	97,425	Human
VP6	I2	OR270779	OVR762	EF554152	98,156	Sheep
			SR294	OR270790	95,725	Roe deer
VP1	R2	OR270775	PR1300	KP198639	98,233	Human
			GA471	OR270742	97,262	Chamois
VP2	C2	OR270776	Hun5	EF554105	97,048	Human
			JE295	OR270765	96,442	Red deer
VP3	M2	OR270777	JE295	OR270766	96,57	Red deer
			GA471	OR270744	96,49	Chamois
NSP1	A11	OR270770	JE295	OR270759	96,477	Red deer
			JE282	OR270748	96,07	Red deer
NSP2	N2	OR270771	PR1300	KP198643	98,741	Human
			JE295	OR270760	98,323	Red deer
NSP3	T6	OR270772	BP1879	FN665684	95,391	Human
			PR1973	KP198655	95,279	Human
NSP4	E2	OR270773	182-02	KU508378	93,928	Human
			OVR762	EF554157	92,22	Sheep
NSP5	H3	OR270774	AS970	KU317465	97,976	Human
			Tottori-SG	AB853900	97,47	Cow

Supplementary Table 13. Whole genome nucleotide comparison to representative strains from Genbank based on percents of nucleotide identity for strain SR294

Gene	Strain SR294		Strains in the Genbank with the closest nucleotide identity based on phylogenetic analysis			
	Genotype	Accession no.	Strain	Accession no.	Nt. Identity (%)	Host
VP7	G10	OR270791	SR333	OR270802	99,438	Roe deer
			SR338	OR270813	96,629	Roe deer
VP4	P[15]	OR270789	D38-14	KU708257	98,777	Roe deer
			SR333	OR270800	98,498	Roe deer
VP6	I2	OR270790	SR333	OR270801	98,826	Roe deer
			D38-14	KU708258	97,318	Roe deer
VP1	R2	OR270786	SR333	OR270797	98,615	Roe deer
			PA169	EF554126	94,667	Human
VP2	C2	OR270787	SR333	OR270798	98,959	Roe deer
			BP1879	FN665678	98,145	Human
VP3	M2	OR270788	174-1	KF500209	95,273	Pig
			SR338	OR270810	93,398	Roe deer
NSP1	A3	OR270781	SR338	OR270803	96,934	Roe deer
			D110-15	KY426803	96,325	Roe deer
NSP2	N2	OR270782	2012841174	KJ411439	96,96	Human
			KF17	JF421982	93,291	Human
NSP3	T6	OR270783	D38-14	KU708264	99,035	Roe deer
			PA169	EF554134	96,356	Human
NSP4	E2	OR270784	2012841174	KJ411441	97,533	Human
			Tottori-SG	AB853899	96,395	Cow
NSP5	H3	OR270785	SR338	OR270807	98,145	Roe deer
			PR1300	KP198646	97,302	Human

Supplementary Table 14. Whole genome nucleotide comparison to representative strains from Genbank based on percents of nucleotide identity for strain SR333

Gene	Strain SR333	Strains in the Genbank with the closest nucleotide identity based on phylogenetic analysis

	Genotype	Accession no.	Strain	Accession no.	Nt. Identity (%)	Host
VP7	G10	OR270802	SR294	OR270791	99,438	Roe deer
			SR338	OR270813	96,987	Roe deer
VP4	P[15]	OR270800	D38-14	KU708257	99,378	Roe deer
			SR294	OR270789	98,498	Roe deer
VP6	I2	OR270801	SR294	OR270790	98,826	Roe deer
			111-05-27	EF554141	97,234	Human
VP1	R2	OR270797	SR294	OR270786	98,615	Roe deer
			PA169	EF554126	95,097	Human
VP2	C2	OR270798	SR294	OR270787	98,959	Roe deer
			BP1879	FN665678	98,24	Human
VP3	M2	OR270799	GA471	OR270744	99,481	Chamois
			JE295	OR270766	98,484	Red deer
NSP1	A3	OR270792	GA471	OR270737	99,593	Chamois
			SR338	OR270803	96,68	Roe deer
NSP2	N2	OR270793	GA471	OR270738	99,476	Chamois
			PR1300	KP198643	98,741	Human
NSP3	T6	OR270794	JE295	OR270761	98,499	Red deer
			B10925	EF554123	98,285	Human
NSP4	E2	OR270795	GA471	OR270740	99,62	Chamois
			PR1300	KP198645	97,533	Human
NSP5	H3	OR270796	GA471	OR270741	99,831	Chamois
			PR1300	KP198646	99,325	Human

Supplementary Table 15. Whole genome nucleotide comparison to representative strains from Genbank based on percents of nucleotide identity for strain SR338

Gene	Strain SR338		Strains in the Genbank with the closest nucleotide identity based on phylogenetic analysis			
	Genotype	Accession no.	Strain	Accession no.	Nt. Identity (%)	Host
VP7	G10	OR270813	SR333	OR270802	96,987	Roe deer
			SR294	OR270791	96,629	Roe deer
VP4	P[15]	OR270811	SR333	OR270800	95,086	Roe deer
			D38-14	KU708257	95,064	Roe deer
VP6	I2	OR270812	PA169	EF554130	98,407	Human
			JE295	OR270768	97,318	Red deer
VP1	R2	OR270808	R1WTA17	OL988985	94,046	Cow
			SR333	OR270797	93,537	Roe deer
VP2	C2	OR270809	BP1879	FN665678	97,767	Human
			SR333	OR270798	96,991	Roe deer
VP3	M2	OR270810	174-1	KF500209	94,017	Pig
			SR294	OR270788	93,398	Roe deer
NSP1	A3	OR270803	SR294	OR270781	96,934	Roe deer
			D110-15	KY426803	96,816	Roe deer
NSP2	N2	OR270804	R1WTA17	OL988989	95,388	Cow
			KF17	JF421982	89,413	Human
NSP3	T6	OR270805	Chubut	FJ347108	96,463	Guanaco

			Tottori-SG	AB853898	96,034	Cow
NSP4	E2	OR270806	D38-14	KU708265	97,154	Roe deer
			BP1062	FN665697	95,825	Human
NSP5	H3	OR270807	PR1300	KP198646	98,145	Human
			SR294	OR270785	98,145	Roe deer

Supplementary Table 16: Number of raw reads per each sample

Sample	Raw reads
GA_471	23823686
JE_282	27568666
JE_295	22577400
SR_100	25082984
SR_294	39613810
SR_333	26492330
SR_338	41981736

Supplementary Table 17: Primers for filling gaps in the genome sequence.

Name	Sequence (5' – 3')	Product Size (bp)
JE282_VP1_3,138 R	CGCAACGATGTGATATTCCAC	682
JE282_VP1_2,457 F	GACGTGCACAGATATCGGCA	682
JE282_VP1_660 R	AGAATTGACATTGGTGACGAAACT	660
JE282_VP1_1 F	CAATGAACTGGAAAGTAGATGTATTGA	660

SR333_VP6_1,400 R	TCTCACTACGCCATCTGAGTG	599
SR333_VP6_802 F	ACGGAGCGACTACATGGTAC	599
SR333_VP4_745 F	AGAAGAGCCGCAGTAAACGA	638
SR333_VP4_1,382 R	ATTGTTTGGATTGGCTGCCG	638
SR333_VP4_940 F	GAAGTAACCGCGCACACAAC	719
SR333_VP4_1,658 R	CATGACATCTGTGGCCATCG	719
SR333_VP4_382 F	ACAGCGAGTATATCAGTGGCA	559
SR333_VP4_940 R	CCTCTCCATCGCGCACATAT	559

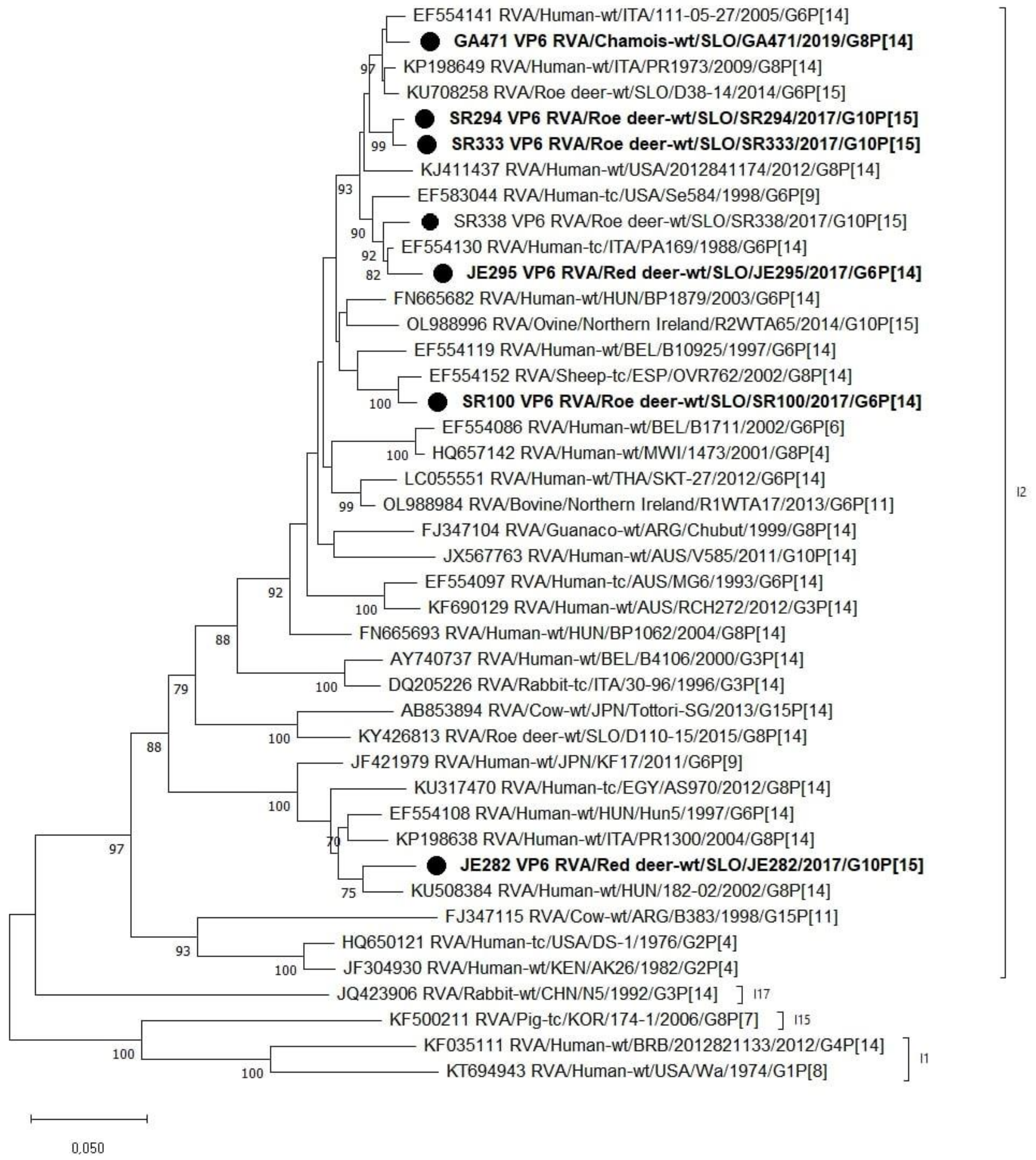

Supplementary Figure 1: The Maximum likelihood phylogenetic tree on VP6 segment. Bootstrap values lower than 70 are not shown. The Slovenian wild ruminants' strains are marked with circle.

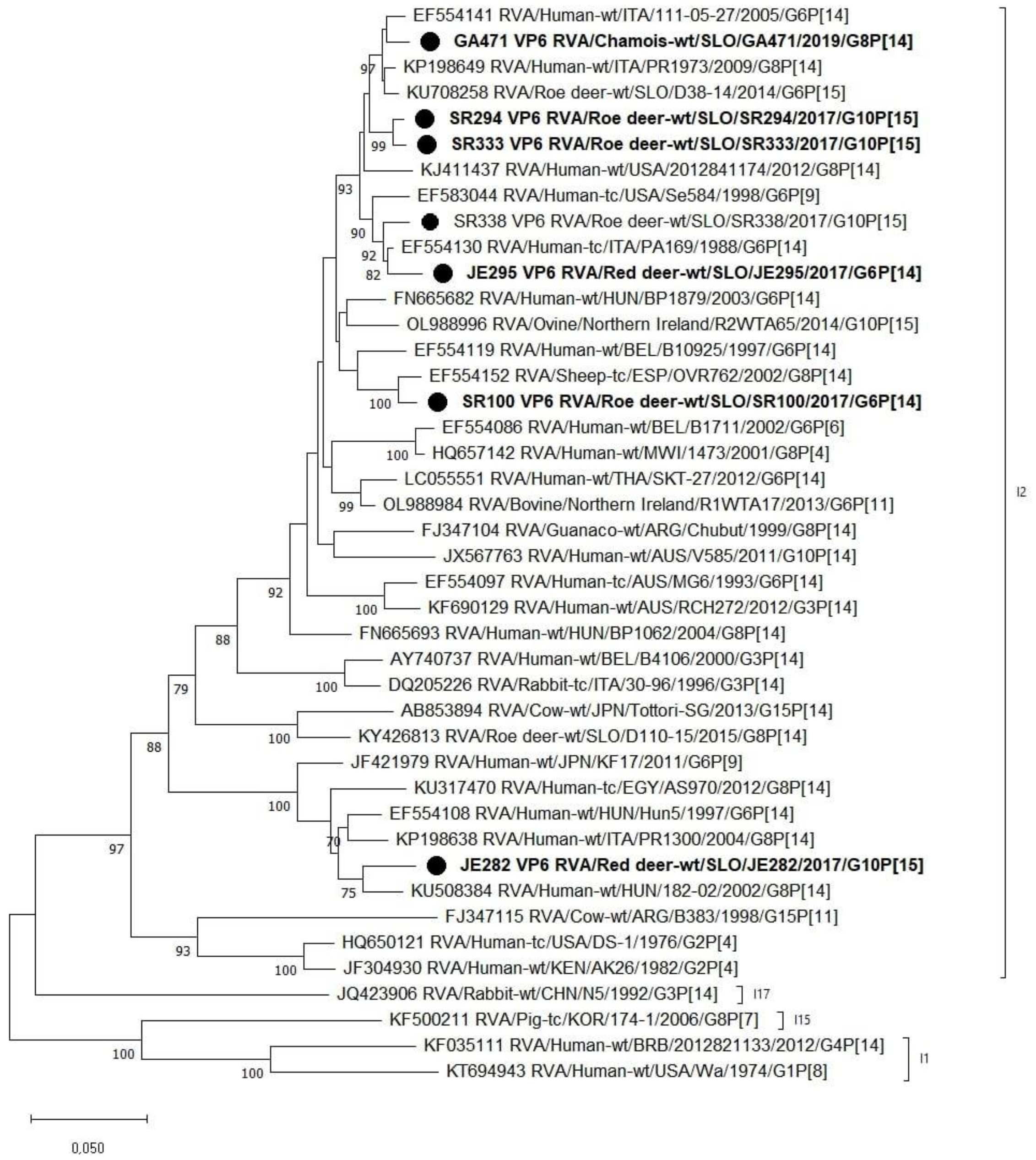

Supplementary Figure 2: The Maximum likelihood phylogenetic tree on VP1 segment. Bootstrap values lower than 70 are not shown. The Slovenian wild ruminants' strains are marked with circle.

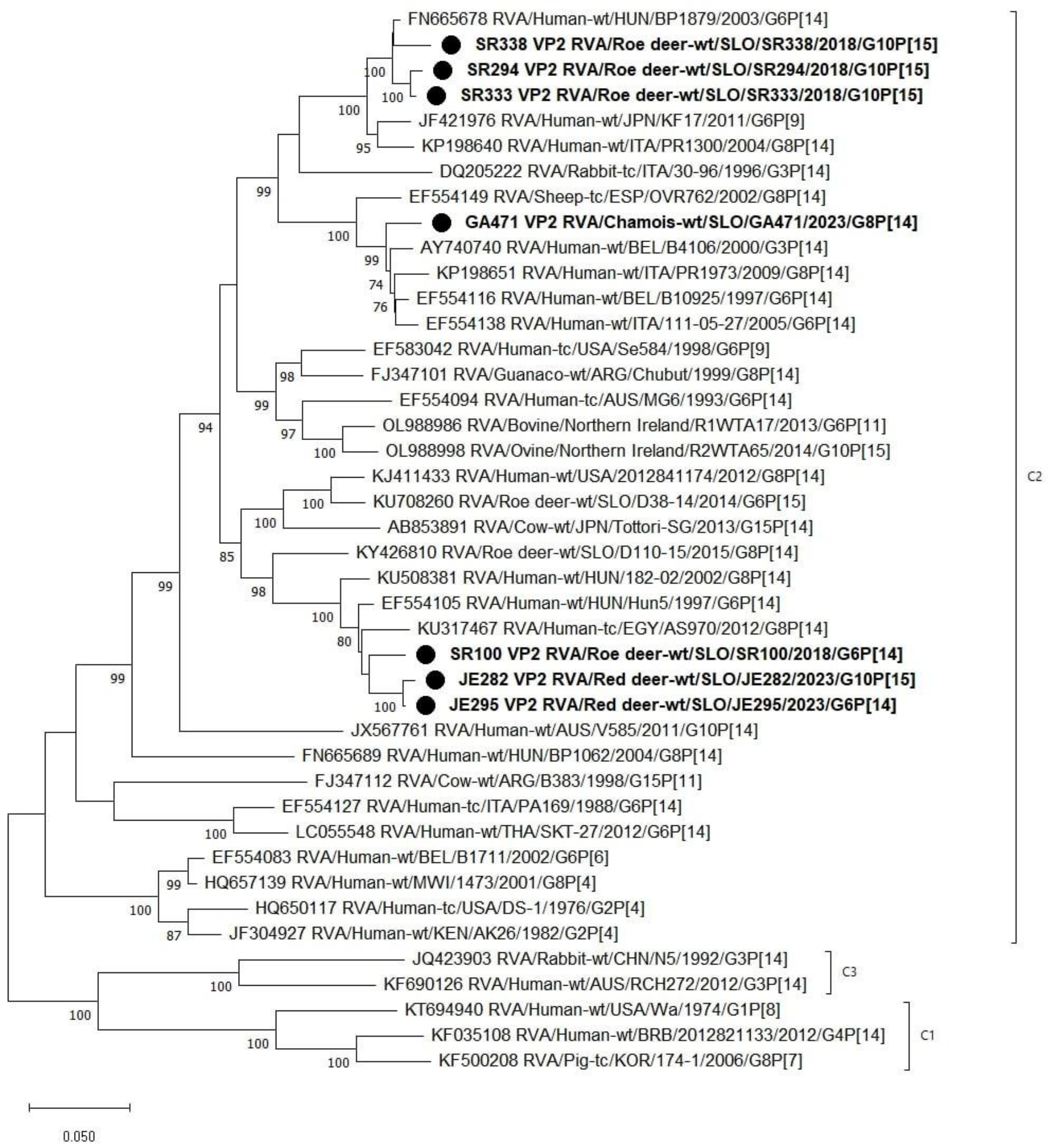

Supplementary Figure 3: The Maximum likelihood phylogenetic tree on VP2 segment. Bootstrap values lower than 70 are not shown. The Slovenian wild ruminants' strains are marked with circle.

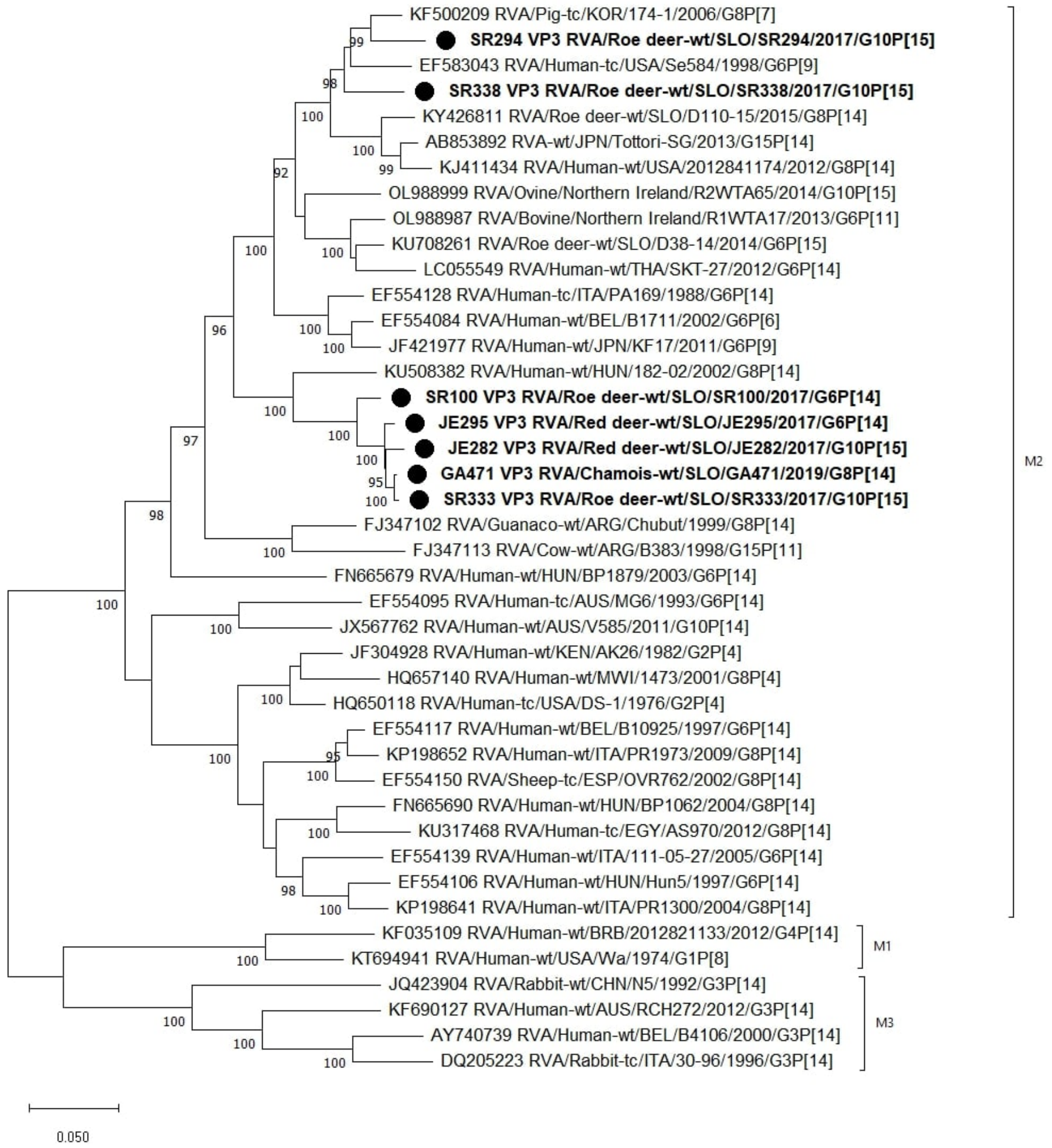

Supplementary Figure 4: The Maximum likelihood phylogenetic tree on VP3 segment. Bootstrap values lower than 70 are not shown. The Slovenian wild ruminants' strains are marked with circle.

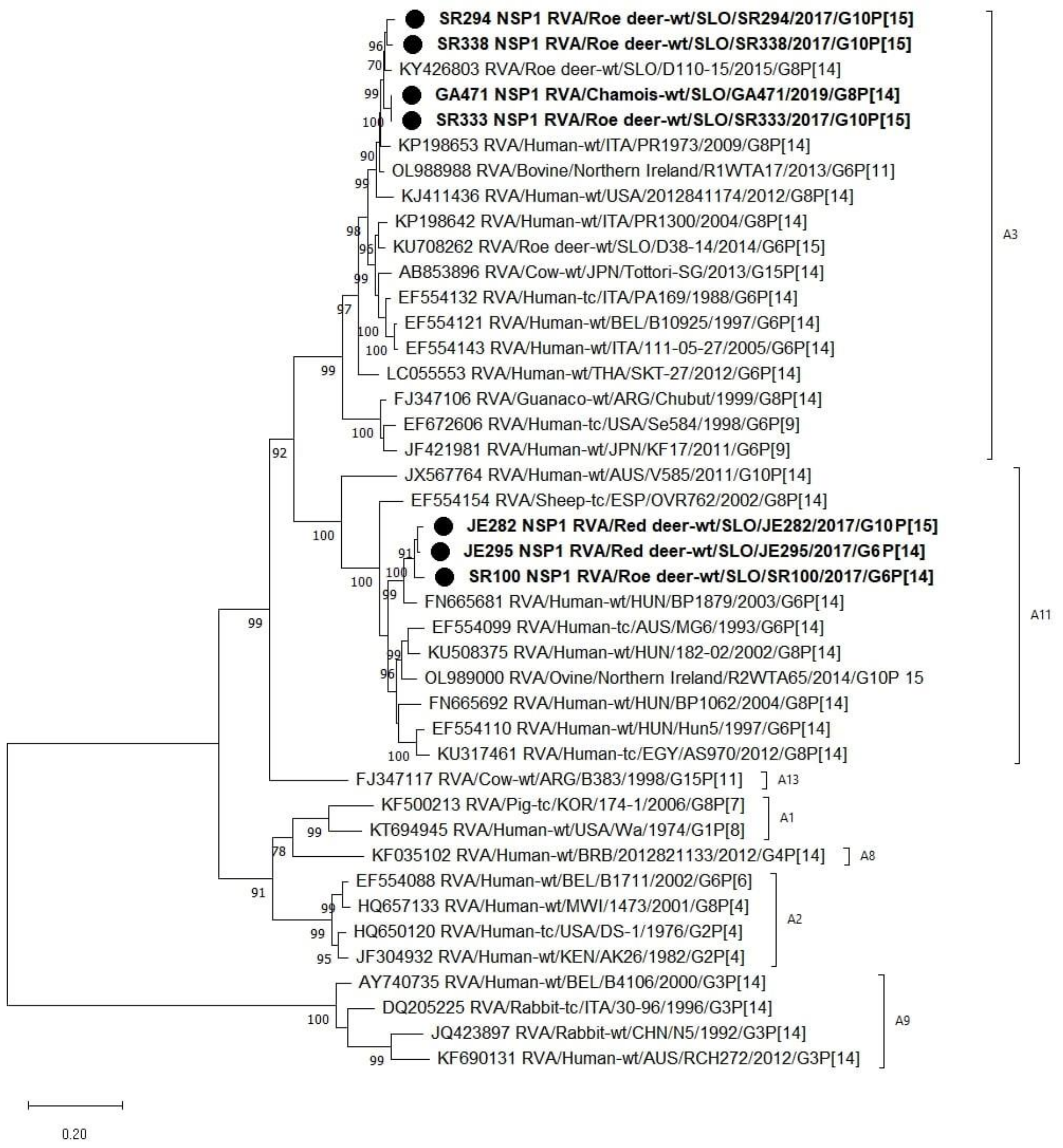

Supplementary Figure 5: The Maximum likelihood phylogenetic tree on NSP1 segment. Bootstrap values lower than 70 are not shown. The Slovenian wild ruminants' strains are marked with circle.

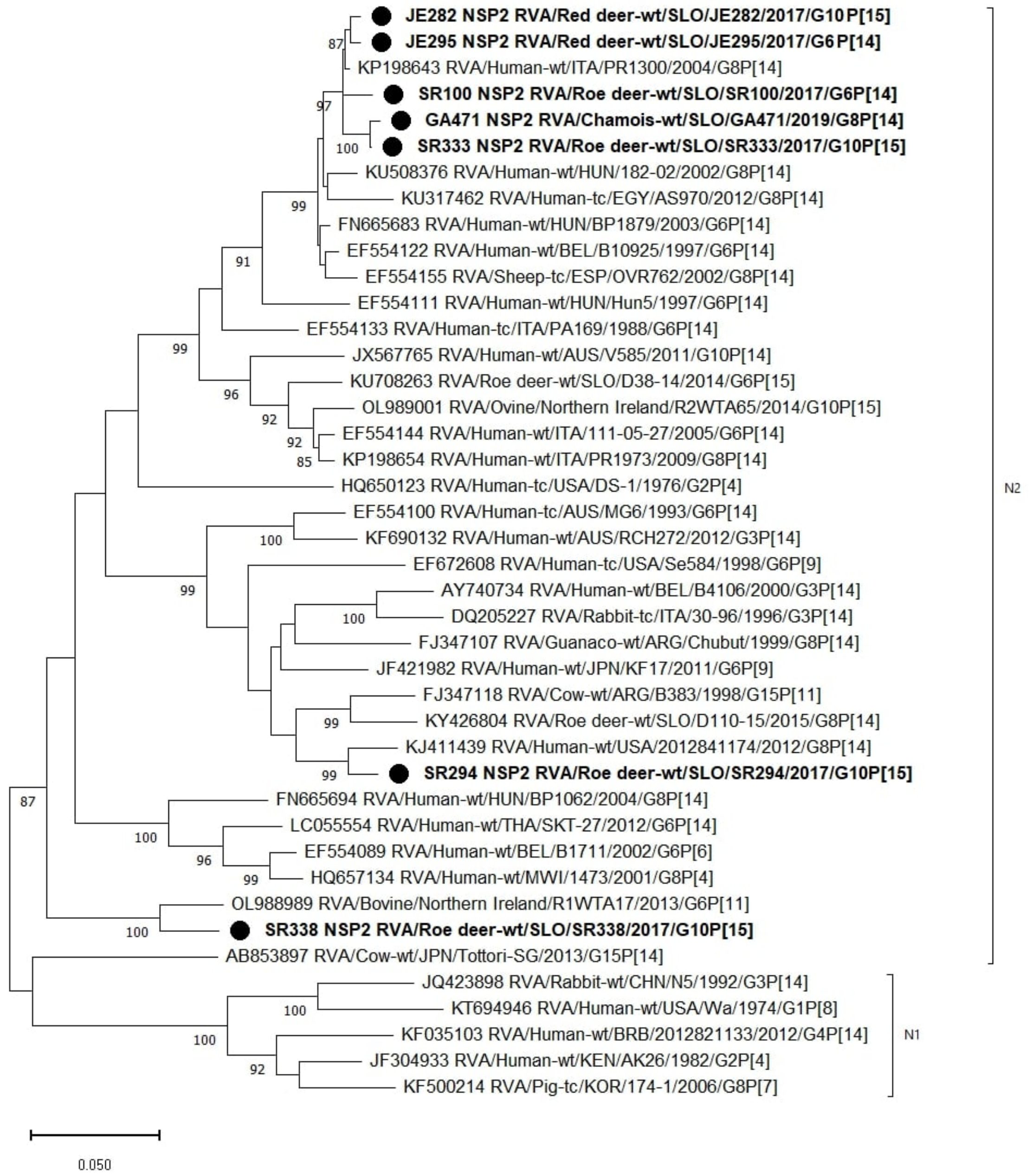

Supplementary Figure 6: The Maximum likelihood phylogenetic tree on NSP2 segment. Bootstrap values lower than 70 are not shown. The Slovenian wild ruminants' strains are marked with circle.

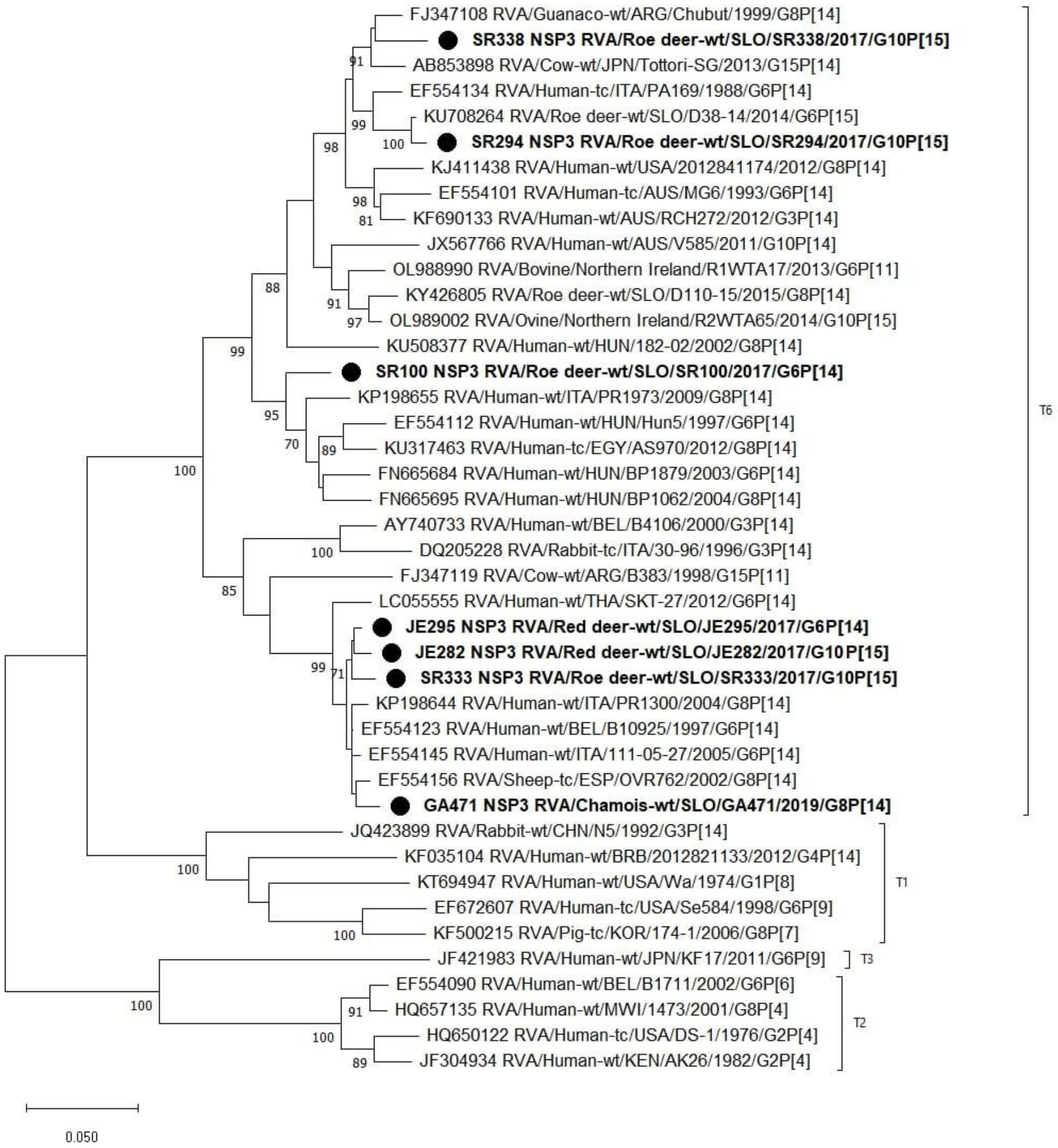

Supplementary Figure 7: The Maximum likelihood phylogenetic tree on NSP3 segment. Bootstrap values lower than 70 are not shown. The Slovenian wild ruminants' strains are marked with circle.

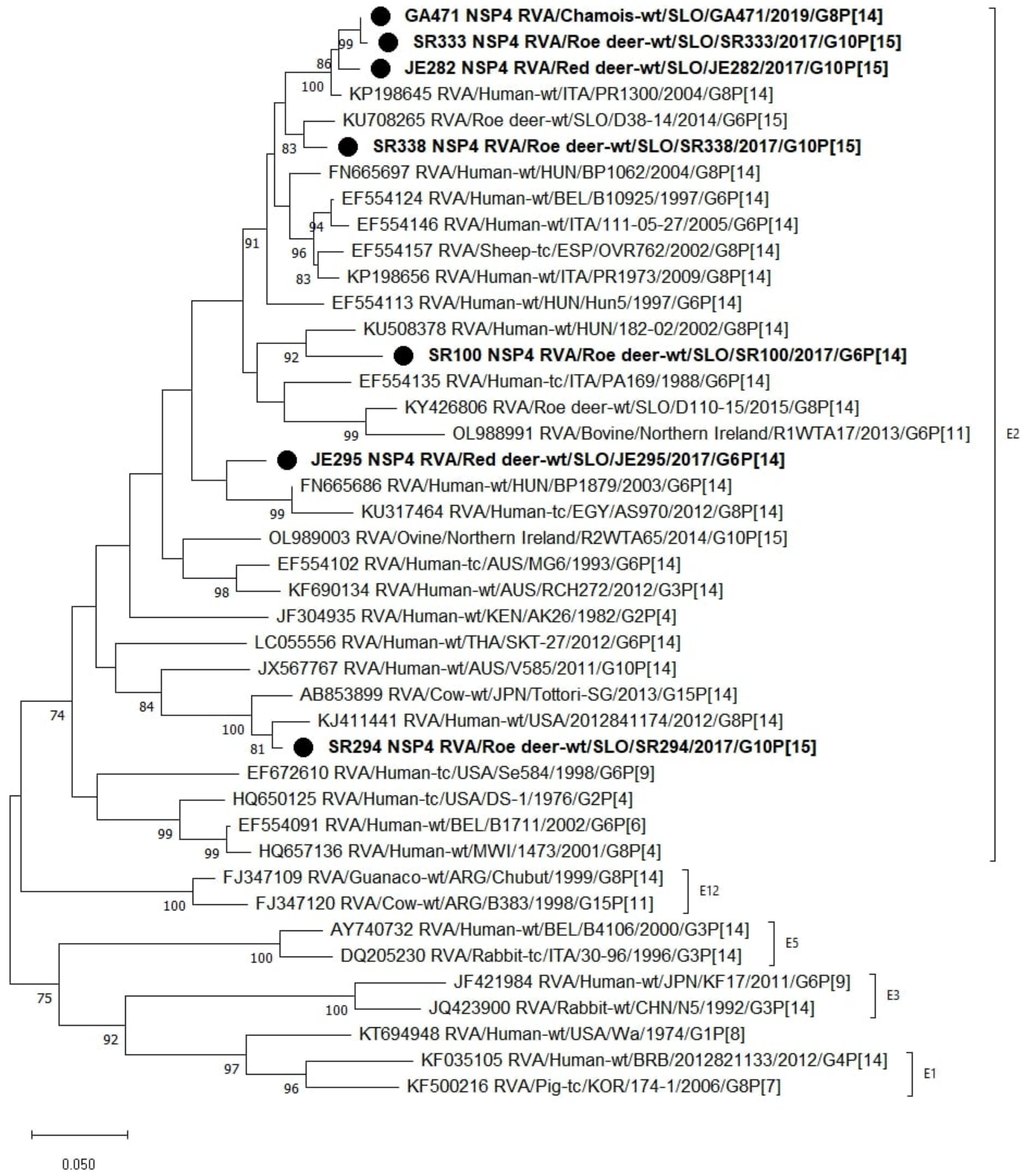

Supplementary Figure 8: The Maximum likelihood phylogenetic tree on NSP4 segment. Bootstrap values lower than 70 are not shown. The Slovenian wild ruminants' strains are marked with circle.

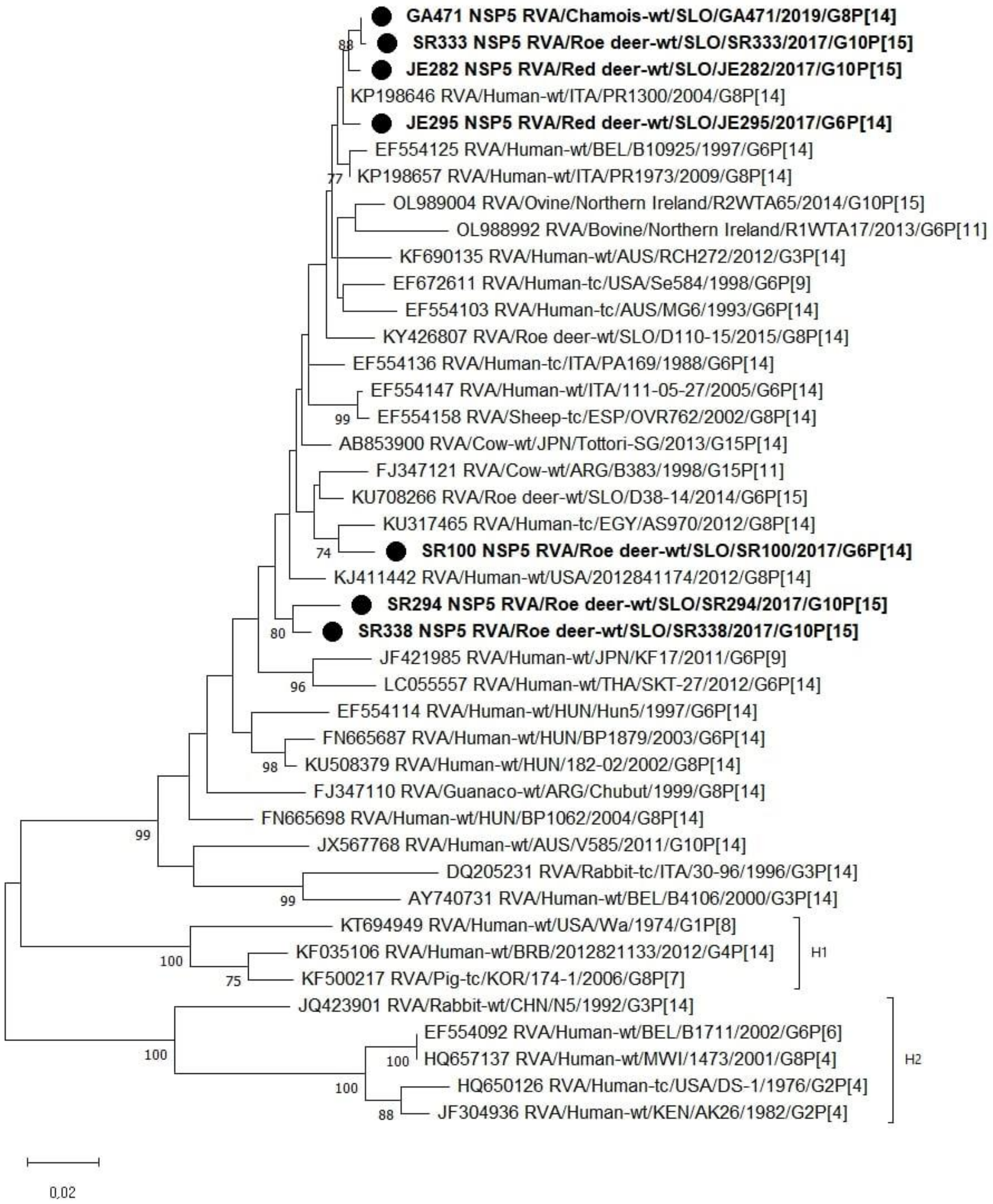

Supplementary Figure 9: The Maximum likelihood phylogenetic tree on NSP5 segment. Bootstrap values lower than 70 are not shown. The Slovenian wild ruminants' strains are marked with circle.
